# Engineering a protein homodimer from a heterodimer: A chimeric DBHS protein

**DOI:** 10.1002/pro.70799

**Published:** 2026-09-21

**Authors:** Andrew C. Marshall, Isabelle Mohnen, Alejandro Villa Gomez, Archa H. Fox, Gavin J. Knott, Charles S. Bond

**Affiliations:** ^1^ School of Molecular Sciences, University of Western Australia Perth Western Australia Australia; ^2^ Fraunhofer Institute for Molecular Biology and Applied Ecology IME Aachen Germany; ^3^ School of Human Sciences, University of Western Australia Perth Western Australia Australia; ^4^ Department of Biochemistry and Molecular Biology, Biomedicine Discovery Institute Monash University Clayton Victoria Australia

**Keywords:** DBHS proteins, Heterodimerisation, NONO, Paraspeckles, protein engineering, protein–protein interactions, RNA binding proteins, SFPQ

## Abstract

Drosophila behavior/human splicing (DBHS) proteins are involved at nearly every stage of the RNA lifecycle. There are three paralogues in mammals—SFPQ, NONO and PSPC1—which form both homodimer and heterodimers. It is likely that the non‐redundant and overlapping roles of the different DBHS paralogues emerge from their dynamic and combinatorial nature; that is, their ability to mix and match dimer partners. A strong preference for heterodimerisation over homodimerisation has been demonstrated. However, the underlying molecular determinants of DBHS partner preference are poorly understood. This study describes the design, and biochemical and structural characterization of a chimeric DBHS protein designed to be a homodimer with an interface that emulates a DBHS heterodimer. In vitro experiments show that this chimera indeed remains as a homodimer even when in the presence of a native DBHS protein that prefers heterodimerisation. The ease of purification, production of high‐quality crystals for X‐ray diffraction, and perfect crystallographic symmetry of this engineered homodimer presents this as an attractive approach for future structural studies on DBHS proteins. This ability to engineer partner preference also highlights the potential for DBHS proteins to serve as versatile building blocks in synthetic biology. Furthermore, comparison of the dimer interface of this engineered homodimer with native DBHS proteins highlights subtle conformational differences in residues at the core of the dimer interface, indicating that maximized packing of a core tryptophan residue combined with increased hydrophobic or polar complementarity at the interface is likely to determine DBHS partner preference.

## 1 INTRODUCTION

The functional complexity of the proteome is greatly expanded by protein–protein interactions, enabling organisms to diversify molecular function without increasing genome size (Babu et al., 2012; Kapoor et al., 2024; Palukuri et al., 2023; Seoane & Carbone, 2021). This principle is exemplified by modular multiprotein assemblies such as transcriptional coactivator complexes (Näär et al., 2001), spliceosomal subunits (Ivanova et al., 2023), and RNA–protein granules (Anderson & Kedersha, 2006), where differential combinatorial pairing of subunits generates distinct functional entities from a limited set of gene products.

In vertebrates, the Drosophila behavior/human splicing (DBHS) protein family is comprised of three paralogues: splicing factor proline/glutamine‐rich (SFPQ), non‐POU domain‐containing octamer‐binding protein (NONO), and paraspeckle protein component 1 (PSPC1). A defining structural feature of DBHS proteins is that they are obligate dimers, with a strong preference for heterodimerisation (Huang et al., 2018; Lee et al., 2022). These multifunctional RNA/DNA‐binding proteins are abundant in the nucleus, participating in a myriad of processes, including transcriptional regulation, pre‐mRNA splicing, RNA transport, DNA double‐strand break repair, and the assembly of paraspeckle biomolecular condensates (Bladen et al., 2005; Fox et al., 2018; Knott, Bond, & Fox, 2016). DBHS proteins have been implicated in innate immunity, oncogenesis and tumor suppression, fertility, and circadian rhythm regulation underscoring their importance across diverse cellular functions (Iannuzzi et al., 2025; Kuwahara et al., 2006; Lahaye et al., 2018; Lang & Jou, 2021; McCluggage & Fox, 2021; Takeiwa et al., 2024; Torres et al., 2017).

**FIGURE 1:**
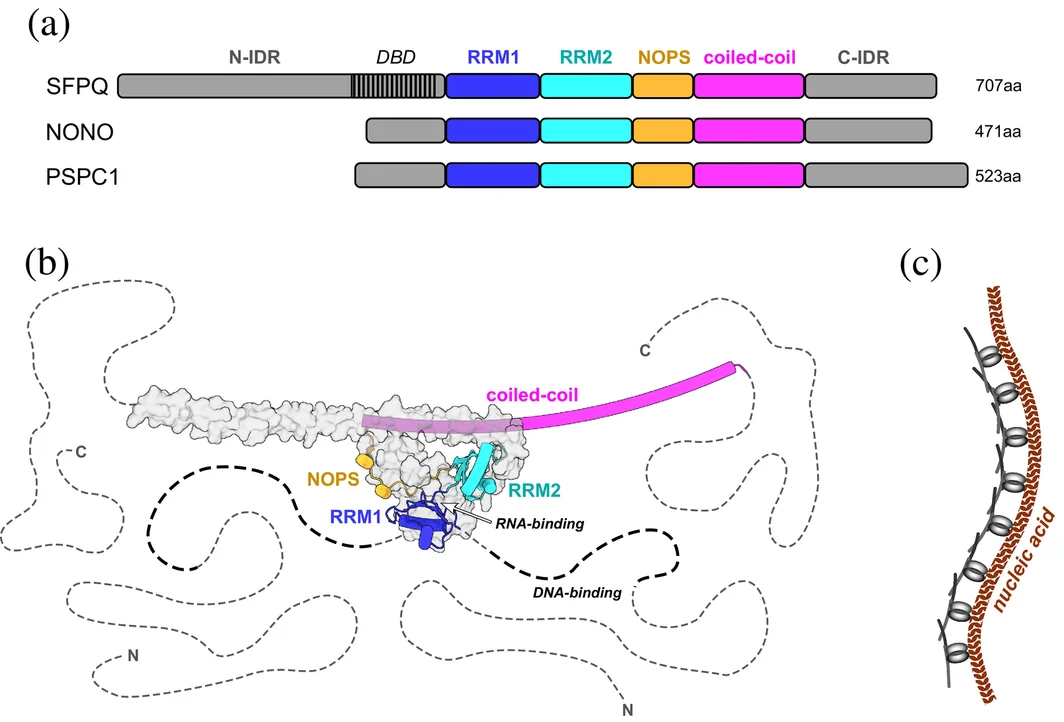
DBHS proteins are obligate dimers that bind nucleic acids. (a) The three human DBHS paralogues contain a highly conserved folded core—composed of tandem RRM domains, a unique NOPS domain, and a coiled‐coil domain—flanked by divergent intrinsically disordered regions (IDRs). The region responsible for DNA‐binding, unique to SFPQ, is indicated by black stripes (‘DBD’). (b) The crystal structure of SFPQ (PDB 4WIJ) is shown as a representative example of DBHS quaternary structure, with α‐helices shown as cylinders and β‐strands as arrows, colored by domain. The surface of the dimeric partner is shown in transparent gray. Gray dashes indicate the flanking N‐ and C‐terminal IDRs (for illustrative purposes only). The DNA‐binding region of SFPQ is immediately N‐terminal of RRM1 (black dashes). The RNA‐binding site is situated on the upper surface of the RRM1 domain (Wang et al., 2022). (c) The extended coiled‐coil α‐helix facilitates inter‐dimer interactions to allow filament formation, leading to a proposed model for the polymerization of DBHS dimers along nucleic acids (Lee et al., 2015; Rasmussen et al., 2025).

DBHS proteins dimerise via a conserved ~300‐residue folded core region comprising tandem RNA recognition motifs (RRM1 and RRM2), a unique NonA/paraspeckle (NOPS) domain, and an extended C‐terminal coiled‐coil (Figure 1) (Huang et al., 2018; Knott et al., 2022; Lee et al., 2015; Lee et al., 2022; Passon et al., 2012). The RNA‐binding specificity of different DBHS proteins is an incompletely resolved question. However, DBHS proteins are known to bind RNA via their RRM domains with broad specificity, recognizing both structured and unstructured targets (Knott et al., 2022; Wang et al., 2022). This lack of specificity is consistent with the very high conservation of residues at the RNA‐binding interface. In addition, SFPQ can bind DNA via part of its intrinsically disordered region N‐terminal to RRM1 (Lee et al., 2015; Urban et al., 2002). Strong evidence supports a model of polymerization along nucleic acid targets via coiled‐coil interactions between neighboring dimers (Figure 1c) (Koning et al., 2024; Lee et al., 2015; Rasmussen et al., 2025). The folded DBHS core is flanked by intrinsically disordered N‐ and C‐terminal regions (IDRs) that vary substantially between paralogues in terms of length and amino acid composition (Koning et al., 2025; Marshall et al., 2023). Different IDRs are likely to facilitate partner‐specific interactions and confer distinct material properties on emergent condensates (Hosokawa et al., 2026; Marshall et al., 2023; Shao et al., 2022; Zhang et al., 2022), underlying DBHS functional diversity. Importantly, all possible dimeric combinations (both homo‐ and heterodimers) occur between the three different DBHS paralogues, making possible six distinct protein species from only three different gene products (excluding splice isoforms), expanding protein functionality without expanding the genome.

Although DBHS proteins exist as homodimers in isolation, they have a strong preference for heterodimerisation in the presence of other paralogues (Huang et al., 2018; Lee et al., 2022). This is consistent with a selective pressure for increased functionality via hetero‐interactions. It is likely that this modularity contributes to their capacity for facilitating a huge diversity of intracellular processes (Knott, Bond, & Fox, 2016). Subtle sequence and conformational differences at the dimer interface have been proposed to underlie paralogue‐specific pairing preferences (Huang et al., 2018; Knott et al., 2022; Laurenzi et al., 2022), but a definitive structural explanation for heterodimer preference is lacking.

Here, we employ a novel approach for investigating DBHS dimers by engineering a chimeric DBHS protein which, as a homodimer, is designed to have an interface that mimics the SFPQ‐NONO heterodimer interface. We show that, in contrast to native human DBHS proteins, the chimera remains as a homodimer in the presence of other DBHS dimers. This chimeric DBHS homodimer retains interaction with a known DBHS nucleic acid substrate, is straightforward to purify and readily forms quality crystals for atomic resolution structural analyses. Comparative analysis with existing DBHS dimer structures reveals specific interfacial residues and structural features that likely dictate the strong heterodimer preference observed in the native proteins. These findings provide new mechanistic insight into DBHS dimerisation specificity and its role in the functional versatility of this protein family.

## 2 RESULTS

### 2.1 Design and production of a chimeric DBHS protein homodimer

**FIGURE 2:**
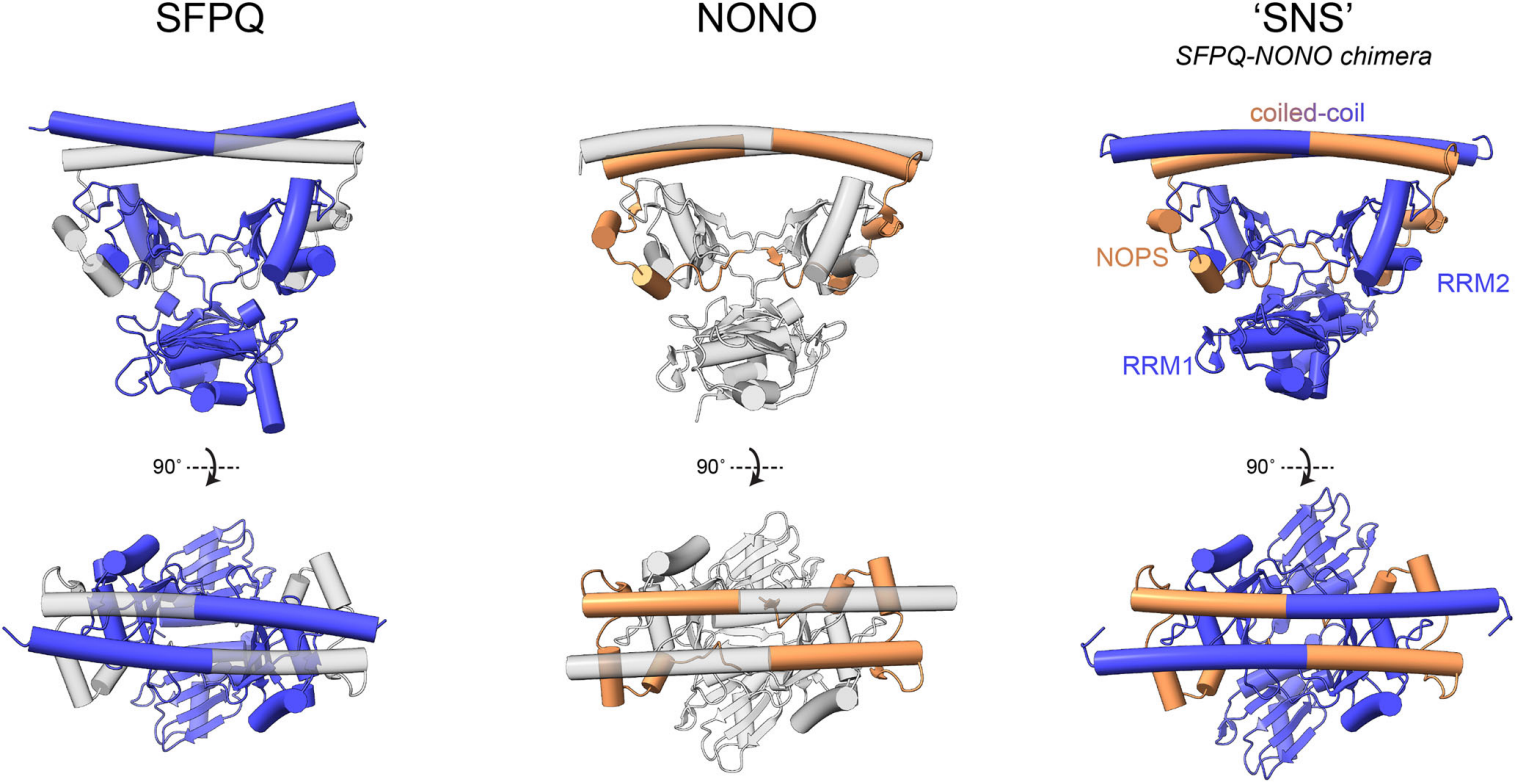
An SFPQ‐NONO‐SFPQ chimeric homodimer (SNS) was constructed by combining the RRM domains and the C‐terminal half of the coiled‐coil of SFPQ (blue; left; PDB ID 4WII) with the NOPS domain and N‐terminal half of the coiled‐coil of NONO (orange; centre; PDB ID 5IFM). The asymmetric unit of the SNS X‐ray crystal structure (right; PDB ID 7LRU) contains only one molecule; its dimeric partner (also displayed) is related by crystallographic two‐fold rotational symmetry.

All six human DBHS dimer combinations have been well described in previous structural studies, showing that the minimal dimer interface is composed of residues from RRM2, the NOPS domain and the coiled‐coil (Huang et al., 2018; Knott et al., 2022; Lee et al., 2015; Lee et al., 2022; Passon et al., 2012; Schell et al., 2022). The NOPS domain of one partner wraps around RRM2 of the other partner, followed by an antiparallel coiled‐coil, resulting in an intimately intertwined dimer. It follows that specific residues at this interface determine DBHS dimer partner preference. We therefore replaced a region encompassing the NOPS domain and part of the coiled‐coil (amino acids 453–504) of SFPQ with the corresponding region of NONO (230–281) to produce a chimeric DBHS protein with a dimerisation interface resembling one half of the SFPQ‐NONO heterodimer interface (Figure 2). This 52 residue‐long replacement includes 14 residues that are not conserved between SFPQ and NONO. That is, this SFPQ‐NONO‐SFPQ chimeric protein is equivalent to SFPQ with 14 substitutions. The termini were selected based on previous crystallization and in vitro nucleic acid binding studies on DBHS protein truncations (Knott et al., 2022; Lee et al., 2015). The final protein is 260 amino acids long, composed of SFPQ(276–452) + NONO(230–281) + SFPQ(505–535), encompassing both RRM domains, the NOPS domain, and the coiled‐coil. Herein, we refer this truncated chimeric protein as ‘SNS’.

Using a previously established protocol (see Materials & Methods), purification of recombinant SNS from *E. coli* culture was straightforward, with no visible protein precipitation occurring, even when concentrating to 16 mg/mL. Qualitatively, this is in contrast with the relatively more difficult purification of equivalent protein truncations of wildtype DBHS proteins, particularly for NONO, which is prone to precipitation during purification and concentration (Knott, Panjikar, et al., 2016), hinting that SNS is more a more soluble, less aggregation‐prone protein in vitro.

To confirm the functionality of our DBHS chimera we measured its ability to bind a known DBHS substrate. Previous studies have shown that NEAT1_2 antisense oligonucleotides with a phosphorothioate‐modified backbone (PS‐ASO) bind DBHS proteins in vivo and in vitro (Knott et al., 2022; Shen et al., 2015). One such PS‐ASO, IONIS742093, is a 20‐mer modified at the 2′‐ribose position with an α‐fluoro moiety. As this is the highest affinity nucleic acid for a DBHS protein determined to date, it makes a suitable and tractable control for the nucleic acid binding function of DBHS proteins. We determined the dissociation constant, *K*_D_, of 105 nM for the interaction of IONIS742093 with SNS (Figure S2), showing that SNS retains high‐affinity interaction with a known nucleic acid substrate of DBHS proteins. This binding affinity is comparable with that determined previously for the IONIS742093 interaction with the NONO homodimer of 40.8 nM (Knott et al., 2022).

### 2.2 SNS remains as a homodimer in the presence of SFPQ

A preference for heterodimerisation over homodimerisation is well established for the human DBHS paralogues (Bladen et al., 2005; Fox et al., 2005; Huang et al., 2018; Lee et al., 2022). Lee et al. (2022) used affinity chromatography and analytical gel‐filtration experiments to demonstrate that when SFPQ and NONO homodimers are incubated together in vitro, the SFPQ‐NONO heterodimer emerges as the predominant species. Given that, as a homodimer, SNS should have an interface equivalent two preferred SFPQ‐NONO half‐interfaces related by two‐fold rotational symmetry, we hypothesized that it would have a preference to remain homodimeric, even in the presence of other DBHS proteins.

**FIGURE 3:**
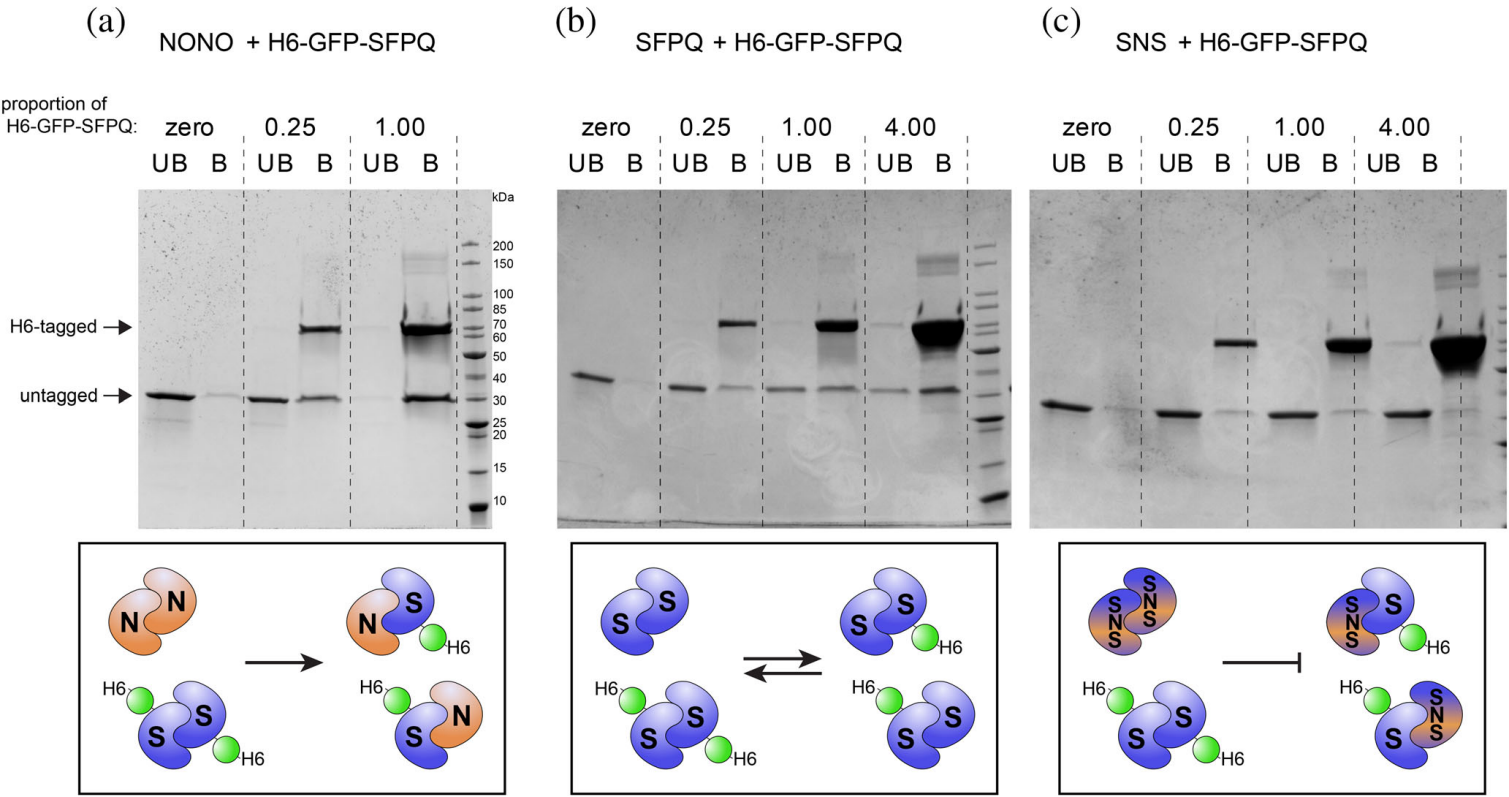
SFPQ readily undergoes partner exchange with (a) NONO and (b) other SFPQ molecules, but not with (c) SNS. For each experiment, an SFPQ fusion protein containing a 6 × histidine + GFP tag (H6‐GFP‐SFPQ) was mixed at increasing molar proportions with untagged NONO, SFPQ or SNS and subjected to Ni^2+^‐affinity chromatography. Samples showing proteins that do not bind the Ni^2+^‐NTA resin (‘unbound’, UB) and those that bind the resin (‘bound’, b) are indicated. In the absence of His‐tagged protein, untagged proteins do not bind the Ni^2+^‐NTA resin (left lanes). For native DBHS proteins (a and b), the unbound amount decreases as tagged SFPQ is added, indicating an interaction. In contrast, SNS (c) remains unbound, even in the presence of four‐fold molar excess tagged SFPQ.

To test this, we mixed pairs of purified DBHS proteins and subjected the mixture to Ni^2+^‐affinity chromatography. Four different protein truncations were assayed (Figure S1): (1) an SFPQ truncation (amino acids 276–598) fused at its N‐terminus to a H6‐mEGFP tag, (2) untagged NONO(53–312), (3) untagged SFPQ(276–535), and (4) untagged SNS. All truncations included the conserved domains (i.e. RRM1/2, NOPS and coiled‐coil) that form the dimer core, and only one (the mEGFP‐SFPQ fusion) was fused to a hexahistidine (H6) tag to facilitate binding to the affinity resin. In agreement with Lee et al. (2022), after incubation with tagged SFPQ for 20 min, NONO bound to the resin (Figure 3a). The amount of bound NONO was approximately equal to the amount of tagged SFPQ in the mixture, with complete binding of NONO observed where the mixture contained equimolar SFPQ (lanes 5 and 6), consistent with a strong preference for heterodimer formation. Similarly, when untagged SFPQ was mixed with tagged SFPQ, the amount of untagged SFPQ bound to the resin scaled with the amount of tagged SFPQ included in the mixture (Figure 3b). Half of the untagged SFPQ was bound where the mixture contained equimolar tagged SFPQ. This is consistent with no dimeric preference (i.e. equilibrium constant = 1), as expected. In contrast, tagged SFPQ was unable to induce binding of untagged SNS, even where SFPQ was in four‐fold molar excess (Figure 3c). No increase in binding was observed when the incubation time was increased to 5 h (Figure S3). This confirms that SNS does not readily exchange partners with SFPQ in vitro, but instead maintains a homodimeric configuration, even in the presence of excess SFPQ.

### 2.3 SNS crystal structure

We then screened conditions to assess the crystallisability of SNS protein and gain atomic resolution insight into its observed homodimeric preference. SNS was subjected to crystallization by vapor diffusion using a range of commercial sparse‐matrix screens (Index, Crystal Screen, MIDASplus). Crystals were observed in a range of conditions after 3–5 days. The best of these grew as small plates which diffracted to ~1.6 Å using synchrotron radiation (Table 1). The crystal structure of SNS was solved by molecular replacement in space group *C*222_1_ and refined using data to 1.6 Å (Table 2). The resulting electron density map was of high quality, allowing the positions of all residues to be modeled with high confidence (Figure S4), except for the five residues at the extreme N‐terminus (276–280), for which no clear electron density was observed.

**TABLE 1:** X‐ray crystallographic data collection and processing.

Diffraction source	Australian Synchrotron beamline MX2
Wavelength (Å)	0.95373
Temperature (K)	100
Detector	ADSC quantum 315r CCD
Crystal‐detector distance (mm)	250
Rotation range per image (°)	0.1
Total rotation range (°)	360
Exposure time per image (s)	0.01
Space group	*C*222_1_
*a*, *b*, *c* (Å)	73.67, 107.16, 68.31
α, β, γ (°)	90, 90, 90
Mosaicity (°)	0.08
Resolution range (Å)	45.38–1.60 (1.63–1.60)
Total No. of reflections	468,399 (15470)
No. of unique reflections	36,031 (1786)
Completeness (%)	100.000 (100.000)
Redundancy	13.0 (8.7)
⟨*I*/σ(*I*)⟩	19.4 (1.0)^a^
*R*_meas_	0.055 (2.243)
*R*_p.i.m_	0.015 (0.746)
*CC*_1/2_	1.000 (0.568)
Overall *B* factor from Wilson plot (Å^2^)	32.66

*Note*: Values for the outer shell are given in parentheses.

^a^ Mean *I*/σ(*I*) falls below 2.0 at a resolution of 1.70 Å. High resolution cut‐off for data processing was selected primarily by reference to *CC*_1/2_ as recommended by (Karplus & Diederichs, 2015).

**TABLE 2:** Structure solution and refinement.

Resolution range (Å)	32.42–1.600 (1.657–1.600)
Completeness (%)	99.92 (99.97)
σ cutoff	*F* > 1.340σ(*F*)
No. of reflections, working set	34,144 (3354)
No. of reflections, test set	1842 (192)
Final *R*_cryst_	0.218 (0.366)
Final *R*_free_	0.256 (0.389)
Cruickshank DPI	0.135
No. of non‐H atoms	
Protein	2073
Water	213
Total	2286
R.M.S. deviations	
Bonds (Å)	0.0032
Angles (°)	0.61
Average *B* factors (Å^2^)	
Protein	45.76
Water	48.47
Ramachandran plot	
Most favored (%)	98.42
Allowed (%)	1.58

*Note*: Values for the outer shell are given in parentheses.

The asymmetric unit contains one protein molecule, with its homodimeric partner related by a two‐fold crystallographic symmetry axis (Figure 2). The quaternary structure is essentially the same as for all other DBHS protein crystal structures. The arrangement of the four RRM domains approximates a tetrahedron; the β‐sheets of each RRM1 domain are in approximately the same plane, exposed to solvent, while the RRM2 β‐sheets face one another at the centre of the dimer. The NOPS domain of one partner embraces the RRM2 domain of the other, almost completely encircling it. Finally, the C‐terminal α‐helix (α6) forms a coiled‐coil along one edge of the tetrahedron.

The resolution and content of the asymmetric unit are noteworthy: despite no optimisation of the initial hit condition, this is the highest resolution diffraction observed among the 17 DBHS protein crystal structures reported to date (all other structures were solved using data to a highest resolution of 1.83–3.5 Å); and there is only one other published structure for which the DBHS dimer displays strict crystallographic two‐fold rotational symmetry (Hewage et al., 2019) (all other structures have ≥ two molecules per asymmetric unit; that is, other homodimers exhibit *non*‐crystallographic symmetry) (Table 3).

**TABLE 3:** DBHS protein X‐ray crystal structures deposited at the Protein Data Bank to date.

DBHS dimer	PDB ID	Space group	Unit cell parameters α, β, γ (°); a, b, c (Å)	Polymers in ASU	Refinement resolution (Å)	RRM1 conformation	Citation
SFPQ homodimer	4WII	P 21 21 21	90, 90, 90; 63.447, 67.611, 119.528	2	2.05	Open	Lee et al. (2015)
SFPQ homodimer	4WIJ	P 43 21 2	90, 90, 90; 66.58, 66.58, 398.02	2	3.49	Open+intermediate	Lee et al. (2015)
SFPQ homodimer	4WIK	C 2 2 21	90, 90, 90; 127.399, 180.416, 57.029	2	3	N/A	Lee et al. (2015)
SFPQ homodimer	6NCQ	C 2 2 21	90, 90, 90; 65.131, 119.167, 72.773	1	1.9	Open	Hewage et al. (2019)
SFPQ homodimer	6OWJ	P 1 21 1	90, 95.74, 90; 61.23, 62.45, 67.88	2	1.94	Open	Huang et al. (2020)
SFPQ homodimer	7UK1	P 1 21 1	90, 97.79, 90; 61.451, 64.658, 68.837	2	2.7	Open	Wang et al. (2022)
SFPQ(L534I) homodimer	7SP0	P 1 21 1	90, 96.08, 90; 61.607, 62.717, 67.793	2	1.83	Open	Widagdo et al. (2022)
SFPQ homodimer + RNA	7UJ1	P 3 2 1	90, 90, 120; 186.738, 186.738, 55.372	4	3.5	Closed	Wang et al. (2022)
SFPQ‐NONO	6WMZ	P 21 21 21	90, 90, 90; 66.63, 112.94, 204.62	4	2.85	Open	Koning et al. (2024)
SFPQ‐NONO	7LRQ	P 31 2 1	90, 90, 120; 66.128, 66.128, 203.561	2	2.3	Open+intermediate	Lee et al. (2022)
SFPQ‐NONO	7PU5	C 1 2 1	90, 105.89, 90; 466.999, 66.82, 126.893	12	2.999	Open	Schell et al. (2022)
SFPQ‐PSPC1	5WPA	C 1 2 1	90, 95.06, 90; 89.229, 67.841, 92.204	2	2.29	Open+intermediate	Huang et al. (2018)
SNS homodimer	7LRU	C 2 2 21	90, 90, 90; 73.665, 107.162, 68.305	1	1.6	Closed	*Current study*
PSPC1‐NONO	3SDE	C 1 2 1	90, 99.96, 90; 90.9, 67.18, 94.08	2	1.9	Open+intermediate	Passon et al. (2012)
NONO homodimer	5IFM	P 1 21 1	90, 97.75, 90; 67.15, 407.177, 68.961	12	2.6	Open	Knott et al. (2022)
PSPC1 homodimer	5IFN	P 1 21 1	90, 98.06, 90; 61.536, 63.493, 67.8	2	3.17	Open	Knott et al. (2022)
NONO‐1 (*C. elegans*) homodimer	5CA5	P 1 21 1	90, 101.03, 90; 60.318, 63.115, 83.838	2	2.4	Open	Knott et al. (2015)

**FIGURE 4:**
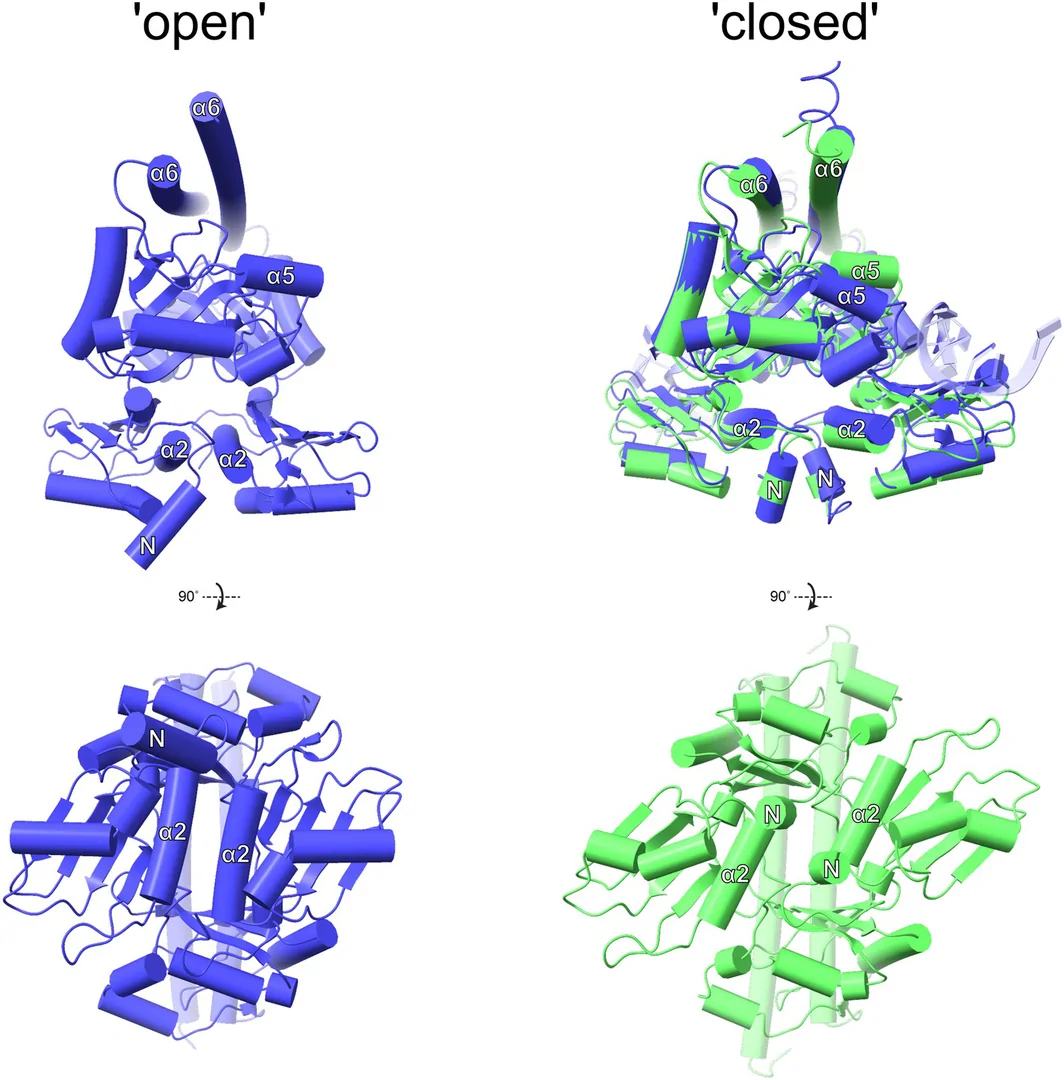
The RRM1 domains of SNS are in a ‘closed’ conformation; a conformation observed previously for the SFPQ‐RNA complex only. Crystal structures of SFPQ (blue) are shown in open (left; 4WII) and closed (right; 7UJ1) conformations. Bound RNA molecules in 7UJ1 are shown as transparent ribbons. Superposition of SNS (green; 7LRU) shows that, despite the absence of bound RNA, the conformation of its RRM1 domains approximates that of the SFPQ‐RNA complex. RRM1 conformational change is coupled to a rearrangement of the RRM1‐RRM1 interface (see bottom view): The α2 helices shift apart, accompanied by the formation of two N‐terminal helices (labeled ‘N’) that pack against one another, contributing a new interface.

Inspection of all published SFPQ‐containing crystal structures (seven homodimers and four heterodimers; see Table 3) highlights the variable pose of the RRM1 domain relative to the dimer core (Figure S5), as observed previously (Hewage et al., 2019; Huang et al., 2018; Knott et al., 2022; Lee et al., 2022; Schell et al., 2022). The repositioning of RRM1 upon nucleic acid binding has been observed previously, where the RRM1 domain rotates towards the dimer core, transitioning from an ‘open’ to a ‘closed’ conformation (Knott et al., 2022; Wang et al., 2022). Of the 11 structures, only one has both RRM1 domains in a closed conformation—this is the only reported crystal structure of a complex between a DBHS protein and RNA (PDB 7UJ1) (Wang et al., 2022). Seven SFPQ‐containing structures fall comfortably into the category of ‘open' RRM1 conformation; the remaining three structures contain RRM1 domains in intermediate conformations (i.e., in‐between closed and open) (Figure S5). Strikingly, despite the absence of RNA in the structure, SNS is most similar to the RNA‐bound SFPQ conformation, with both RRM1 domains approximating a closed conformation (Figure 4). Inspection of the SNS crystal lattice reveals that the C‐terminal end of the α6 helix of a symmetry‐related SNS molecule occupies a large part of the RNA binding pocket, forming extensive contacts with the canonical RNA‐binding surface of RRM1 (Figure S1). The high positive charge present at the RNA binding site has been described (Wang et al., 2022). Accordingly, three acidic residues of the α6 helix insert into the RNA binding pocket. In addition, the two leucine residues at the C‐terminus of SNS diverge from the helix to form a short loop and make extensive contacts with the phenylalanine and arginine residues that form the RRM1 RNA‐binding platform (Figure S6c). It is known that the α6 helix of DBHS proteins extends ~30–60 residues beyond the truncation used in the crystallization experiments here (Koning et al., 2024; Lee et al., 2015; Rasmussen et al., 2025). Therefore, the contacts mediated by these C‐terminal leucine residues are very unlikely to be biologically relevant. It is therefore likely that the closed RRM1 conformation observed here for SNS is induced primarily by lattice effects; an artifact of crystallization. However, via comparison with the SFPQ‐RNA complex, this provides the opportunity to identify conformational effects coupled to RNA‐binding directly versus conformational effects resulting directly from RRM1 conformational change.

In the open conformation, the respective RRM1 domains of DBHS dimeric partners pack against one another via their α2 aliphatic helices (Huang et al., 2018; Knott et al., 2022; Lee et al., 2022). For SFPQ in open conformation, the region immediately N‐terminal of the RRM1 domain is unresolved in most crystal structures, with the exception of two structures where it forms a short α‐helix that is largely solvent‐exposed at the RRM1 periphery (4WII and 6NCQ). In the closed RRM1 conformation, the α2 helices shift ~17 Å apart; the intervening space is filled by the N‐terminal region, which forms a short helix that packs against its counterpart forming a new dimeric interface (Figure 4). Wang and colleagues noted this previously for the SFPQ‐RNA complex; we find that these N‐terminal helices are similarly positioned in the crystal structure of SNS. Altogether, this suggests that remodeling of the region of SFPQ immediately N‐terminal to the RRM1 domain (residues ~280–287) is coupled directly to RRM1 domain conformational change. It has been shown that the affinity of SFPQ for the proto‐oncogene GAGE6 promoter DNA is reduced by the presence of pyrimidine‐rich VL30 retroelement RNA in vitro and in vivo (Song et al., 2005; Wang et al., 2022). Given that the DNA‐binding region of SFPQ is located in the IDR immediately N‐terminal of the RRM1 domain (Figure 1), it is tempting to speculate that the coupling of RRM1 conformation to remodeling of the N‐terminus—suggested here for SNS and previously for SFPQ (Wang et al., 2022)—may point to a potential structural mechanism for allosteric interplay between RNA‐ and DNA‐binding affinity.

**FIGURE 5:**
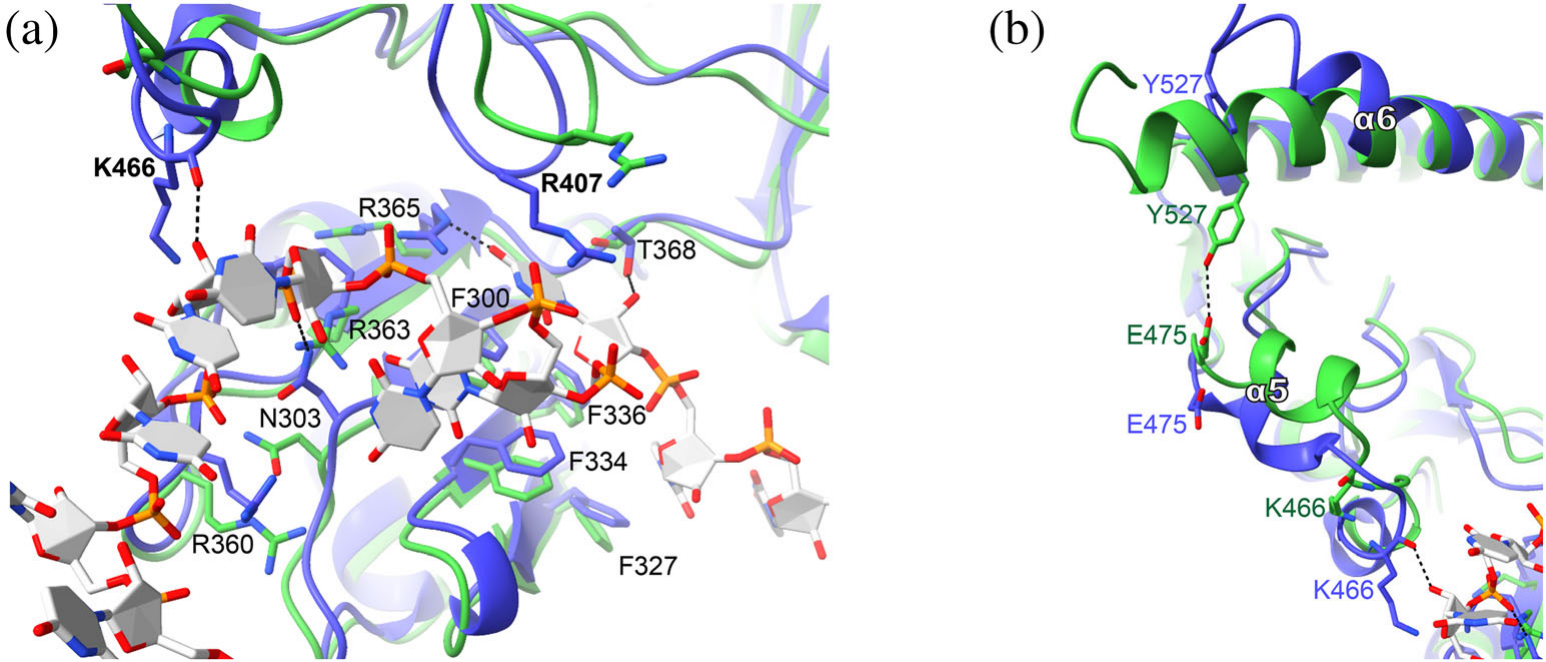
Most RNA‐binding residues are positioned similarly in SNS (green; 7LRU) and SFPQ‐RNA complex (blue; 7UJ1) crystal structures. However, two notable differences indicate RNA‐specific effects: (a) The Arg407 ‘lid’ closes in the presence of RNA to interact electrostatically with RNA backbone phosphates and form a π‐stacking interaction with the central base. (b) The absence of a hydrogen bond between the Lys466 backbone carbonyl and a ribose 2′ hydroxyl groups in SNS results a 3–5 Å upward shift of the α5 helix, allowing direct interaction with Tyr527.

Comparison of SNS and the SFPQ‐RNA complex at the RNA binding site shows that the key residues involved in RNA recognition are in similar positions (Figure 5a). One notable exception is Arg407, which is part of an extended loop in RRM2 and forms a ‘lid’ over the RNA bound across RRM1 via a stacking interaction with the central RNA base (Wang et al., 2022). In the SNS structure, the Arg407 loop is shifted up in a more open conformation, induced by direct hydrogen bonding to the C‐terminal end of the α6 helix of the symmetry‐related SNS molecule (Figure S6c). Adjacent to the RNA binding site, another notable difference between the two structures is the position of the loop preceding the α5 helix in the NOPS domain. The structural plasticity of this region of the NOPS domain—the α5 helix in particular—has been noted previously (Lee et al., 2022). In the SFPQ‐RNA complex this loop interacts directly with RNA via a hydrogen bond between the backbone carbonyl of Lys466 and a ribose 2′ hydroxyl group. In contrast, the SNS structure contains no RNA and therefore no interaction with Lys466. Importantly, this region is unperturbed by the α6 crystal contact. This Lys466 loop and the associated α5 helix are shifted up away from the RNA binding site, allowing direct intermolecular interaction of α5 with the terminal α6 helix of the dimer partner via a Glu475‐Tyr527 hydrogen bond (Figure 5b). This makes possible a mechanism by which SFPQ‐RNA binding could be coupled to α6 conformation via α5. However, the involvement of the α6 helix in crystal packing described above means that, in this case, this interaction is likely to be induced by crystal packing effects, urging us to be cautious about ascribing biological relevance.

### 2.4 Comparison of SNS to SFPQ highlights determinants of dimer partner preference

Given that SFPQ is most like SNS in terms of amino acid content, but has a strong preference for heterodimerisation over homodimerisation, we sought to uncover structural features that confer dimeric partner preference by comparing their respective dimeric structures. There are currently eight published crystal structures of the SFPQ homodimer, encompassing six distinct crystal forms (Table 3). To increase our confidence that observed differences are not the result of different crystal packing, and to reduce the likelihood of effects of poorly modeled sidechains due to low resolution data, we selected a high resolution (~2 Å or better) representative from three different space groups (PDB IDs: 4WII, 6NCQ, 6OWJ). Two of these structures have two molecules in the asymmetric unit, and one has a single molecule in the asymmetric unit, resulting in a total of five unique chains for comparison.

**FIGURE 6:**
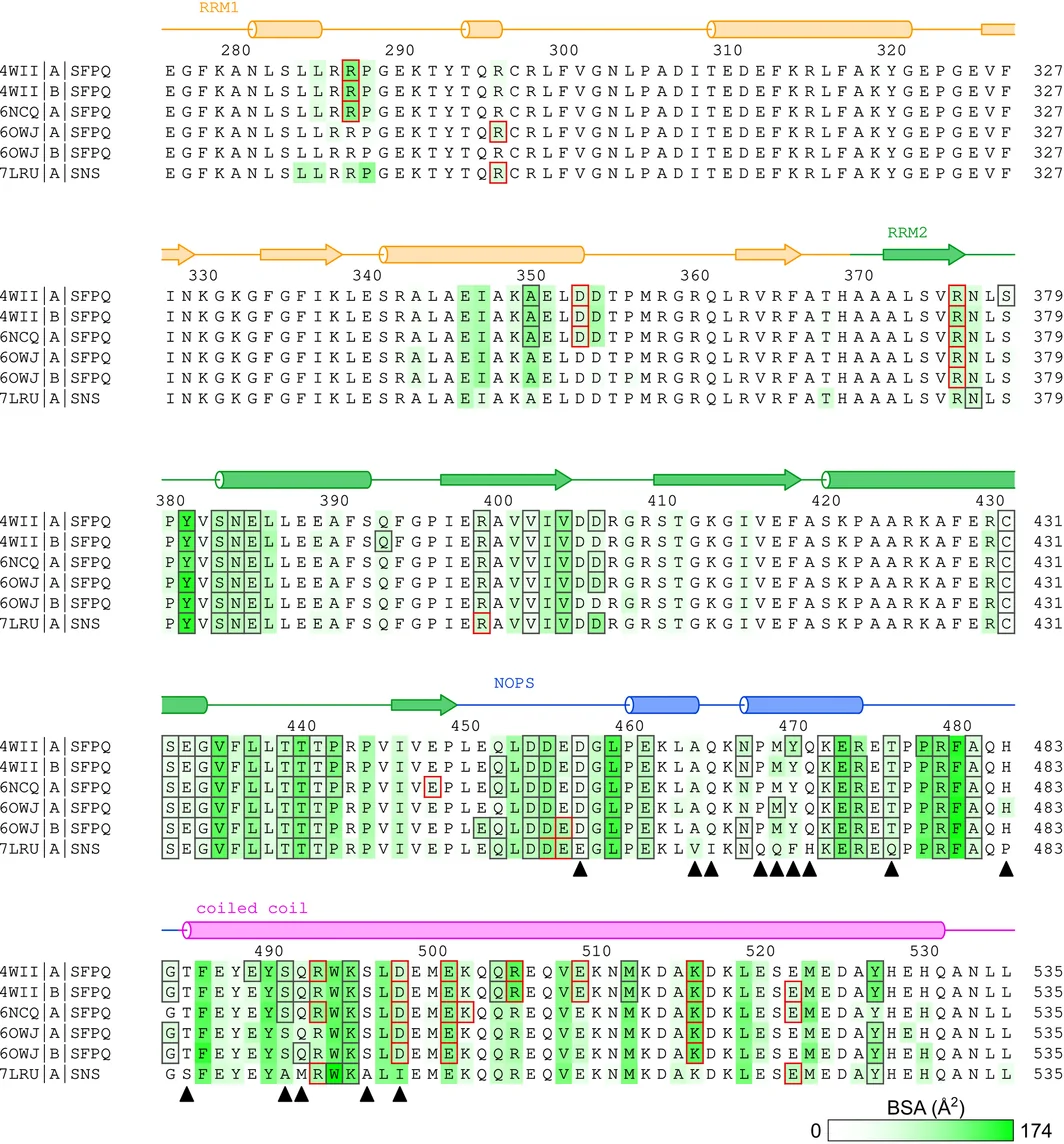
Residues at the dimer interface of SFPQ and SNS identified by *PISA*. An alignment of all five SFPQ chains (three separate crystal structures) and SNS sequences is shown, with residues shaded proportional to their contribution to the dimer interface (‘buried surface area’; BSA). The residue with the greatest BSA across all proteins is Trp494 of SNS (BSA = 174 Å^2^). Residues that contribute hydrogen bonds and salt‐bridges to the dimer interface are framed with black and red boxes, respectively. Black triangles indicate amino acids of SNS that are different to SFPQ (i.e. divergent between NONO and SFPQ). Secondary structural elements corresponding to those present in the SNS crystal structure are shown at top: α‐helices and β‐strands as cylinders and arrows, respectively.

First, we performed protein–protein interface analysis using *PISA* (Krissinel & Henrick, 2007). We found that the estimated solvation free energy gain associated with interface formation was consistently greater for SNS than for all three SFPQ homodimer structures (Table S3), indicating a more hydrophobic dimer interface. To identify residue‐level structural features we then extracted from each *PISA* analysis the surface area contribution of each amino acid residue to the dimeric interface (‘buried surface area’) of SNS and each of the three SFPQ homodimer structures (Figures 6 and S7). Most interfacing residues are in the C‐terminal half of the protein, reflecting the fact that the RRM2, NOPS and coiled‐coil domains form the dimer core. However, the most obvious interface differences between the different structures are in the N‐terminal half of the protein, reflecting the flexibility of the pose of the RRM1 domain, as described in the previous section. Given that we observe this conformational variability of RRM1 between different crystal structures of the same protein, and that RRM1 has been shown to be dispensable for dimer formation (Knott et al., 2022; Lee et al., 2015), it is unlikely that interface differences we observe in this region are informative for determining the basis for dimer partner preference. We therefore excluded RRM1 from the analysis below.

**FIGURE 7:**
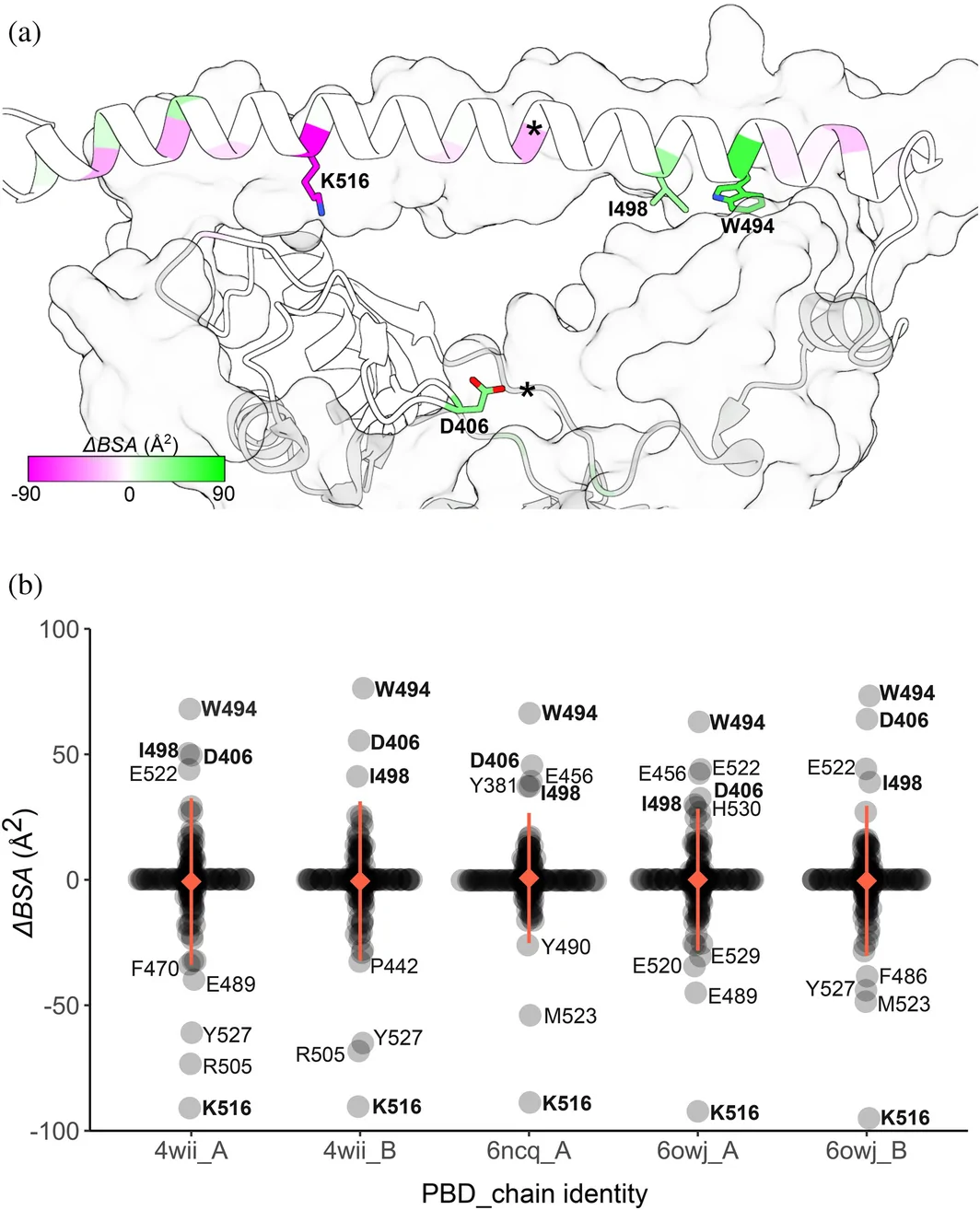
Difference in BSA (ΔBSA) between corresponding SNS and SFPQ residues (excluding RRM1) highlights key residue‐level differences at the dimer interface. More positive ΔBSA values indicate a greater contribution to the dimer interface in SNS compared to SFPQ. (a) SNS crystal structure focussing on the RRM2, NOPS and coiled‐coil, with residues colored by their average difference in BSA (ΔBSA) compared to their corresponding residue in each of the SFPQ chains analyzed. Residues with an absolute average ΔBSA >39 Å are shown as sticks and labeled. Chimeric junctions (i.e., where SFPQ fragments meet NONO NOPS+coiled‐coil fragment) are indicated by asterisks. The dimeric partner is shown in transparent surface representation. (b) Dot plot showing the ΔBSA between SNS and each of the five different SFPQ chains for all residues (excluding RRM1). Residues with a ΔBSA > two standard deviations (red lines) from the average ΔBSA are labeled. Only four residues are consistently identified across all SNS‐SFPQ comparisons (highlighted in bold). These are the same four residues that have the greatest absolute average ΔBSA identified in (a).

To highlight differences at the core dimer interface that may be more subtle, we calculated the *difference* in the buried surface area (ΔBSA) between each of the residues of the RRM2/NOPS/coiled‐coil (residues 370 through 535) of SNS and the respective residue in each of the five SFPQ chains. Most of the residues with the greatest difference are within the coiled‐coil (Figure 7a). Of the residues with the largest differences in buried surface area (> two standard deviations from the mean difference), four are consistently different across all five chains: Asp406, Trp494 and Ile498 all have an increased buried surface area in SNS compared to SFPQ, whereas Lys516 has a lower buried surface area in SNS (Figure 7b).

Asp406 is part of an extended loop in RRM2 and forms an additional weak interchain hydrogen bond with Thr368′ (the prime denotes a residue from the dimeric partner), which is located at the C‐terminal end of RRM1. This contact is absent in all three of the selected SFPQ structures due to the shift of the RRM1 domain downwards, away from the dimer core (Figure S8). Therefore, for Asp406, the observed difference in interface area is due to the conformational flexibility of RRM1, so is unlikely to be a major contributor to dimer partner preference, as discussed above.

**FIGURE 8:**
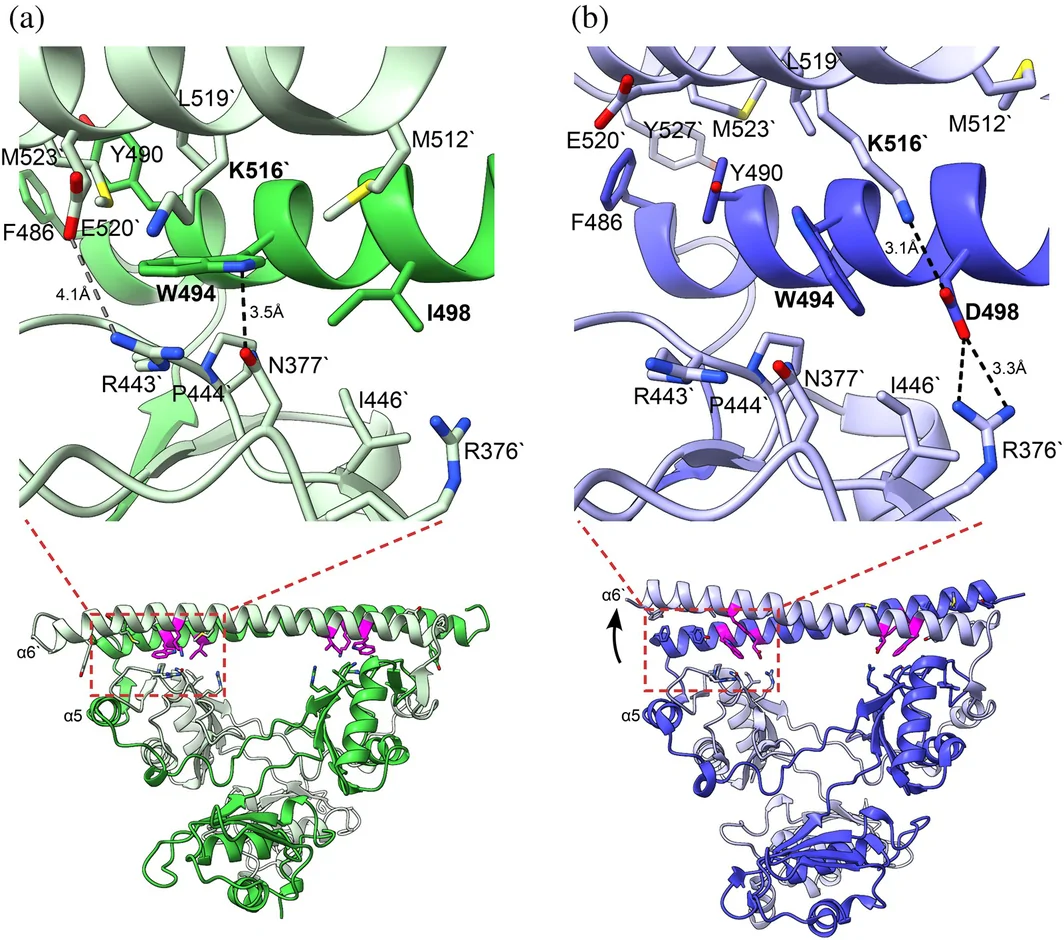
Residues with the greatest difference in BSA between SNS and SFPQ (labeled in bold, and colored magenta in bottom panels) cluster at the core of the dimer interface between the RRM2 domain and coiled‐coil. Black dashes indicate intermolecular hydrogen bonds/salt bridges. (a) The SNS dimer interface is stabilized by Ile498‐Ile446′ and Ile498‐Met512′ hydrophobic interactions, and the insertion of Trp494 between RRM2 (Arg443′, Pro444′) and the α6 helix (Leu519′, Met523′) of the dimeric partner. The closing of Arg443′ (RRM2) and the α6 helix around Trp494 is further stabilized by an intramolecular salt bridge (gray dashes). (b) In contrast, at the SFPQ dimer interface (4WII is shown here), Asp498 engages Lys516′ and Arg376′ in salt‐bridges, and the Trp494 indole is rotated such that its involvement at the dimer interface is reduced. Coupled to this rotation is the repositioning of the α6′ helix further from the underlying RRM2′ domain.

Trp494, Ile498 and Lys516 of SNS are all located in α6 (which forms the coiled‐coil), clustered together at the core dimer interface (Figure 8a). Trp494 and Ile498 are both sandwiched between α6′ and RRM2′ of the dimeric partner, whereas Lys516 points to solvent.

Ile498 forms favorable hydrophobic contacts with Ile446′ (RRM2′) and Met512′ (α6′). Notably, Ile498 is an aspartate residue in native SFPQ, precluding a favorable hydrophobic interaction with Ile446′. In SFPQ, Asp498 instead forms intermolecular salt‐bridges with Arg376′ and Lys516′ (Figure 8b). The involvement of Lys516 in this intermolecular electrostatic interaction in SFPQ but not SNS explains its decreased BSA in SNS (Figure 7).

Trp494 is immediately adjacent to Ile498 in α6, is conserved across DBHS proteins (Knott et al., 2015), and has been shown to be essential for dimerisation and paraspeckle localisation (Passon et al., 2012). It is the bulkiest residue at the dimer interface, contributing a greater BSA to the SNS interface than any other single residue (Figure 6). The indole ring of Trp494 is inserted into the fold of the dimeric partner, between Leu519′/Met523′ (α6′) and Arg443′/Pro444′ (RRM2′) (Figure 8a). The indole ring is in the same plane as α6, such that it forms almost exclusively intermolecular interactions. It makes extensive contacts with atoms along the length of Arg443′, potentially involving favorable π‐alkane, π‐cation and/or π‐π interactions (Crowley & Golovin, 2005; Vedad et al., 2024; Vernon et al., 2018). The favourability of these interactions is evidenced by a shift of Arg443′ 1.2 Å closer to Trp494 compared to its position in SFPQ. The guanidine of Arg443′ forms a weak intramolecular salt bridge with Glu520′, further stabilizing the closing of the RRM2′ domain and α6′ helix around Trp494. In addition, the indole N atom of Trp494 forms a weak hydrogen bond with the amide O atom of Asn377′.

In contrast, in the SFPQ homodimer, the Ile‐Asp substitution at position 498 creates space for the Trp494 indole to rotate ~130° such that it packs against α6, forming relatively more intramolecular interactions, reducing its involvement in the dimer interface (Figure 8b). Indeed, Trp494 is the residue that shows the greatest increase in BSA in SNS compared to all SFPQ structures analyzed (Figure 7b). Furthermore, compared to SNS, the α6 helix of SFPQ is positioned ~2–3 Å further from the underlying RRM2 domain, and appears to be associated with a rearrangement of residues (Tyr490, Phe486, Met523′, Tyr527′) proceeding towards the distal end of the coiled‐coil. This may be necessary to allow for the rotation of Trp494 and collapse of Lys516 to satisfy the salt‐bridge with Asp498.

To investigate whether any of these conformational differences might result from crystal lattice effects, we compared this region of the SNS interface with all three the SFPQ homodimer crystal structures listed above (4WII, 6NCQ, 6OWJ; five distinct chains), and to two different SFPQ‐NONO heterodimer crystal structures (7LRQ and 7PU5; seven distinct dimers). While the conformational flexibility of the C‐terminal end of the α6 helix is apparent, it is shifted similarly for all five SFPQ homodimer chains, further from the underlying RRM2 domain, relative to SNS (Figure S9a). Compared to the SFPQ‐NONO heterodimer structures, SNS contains an additional helical turn at the end of α6, with the overall conformation deviating from the SFPQ α6 in all heterodimers systematically after residue ~523. This is consistent with the significant influence of crystal packing on the SNS C‐terminus, as described in the previous section (Figure S6). However, prior to residue 523, on average, the SFPQ α6 in the SFPQ‐NONO heterodimers more closely approximates the conformation of SNS, suggesting that the conformation of this region is less vulnerable to crystal lattice effects, and more reliably determined by the protein sequence. Indeed, inspection of key residues at the core dimer interface shows that they have very similar conformations in SNS and all SFPQ‐NONO heterodimers (Figure S9c), which are distinct from their conformations in all SFPQ homodimers (Figure S9b). The conformations of residues closer to the distal end of the coiled‐coil (Tyr490, Phe486, Met523′, Tyr527′) are much more variable, consistent with the conformational flexibility of the C‐terminal end of α6, observed here, and in previous structural studies on DBHS proteins.

**FIGURE 9:**
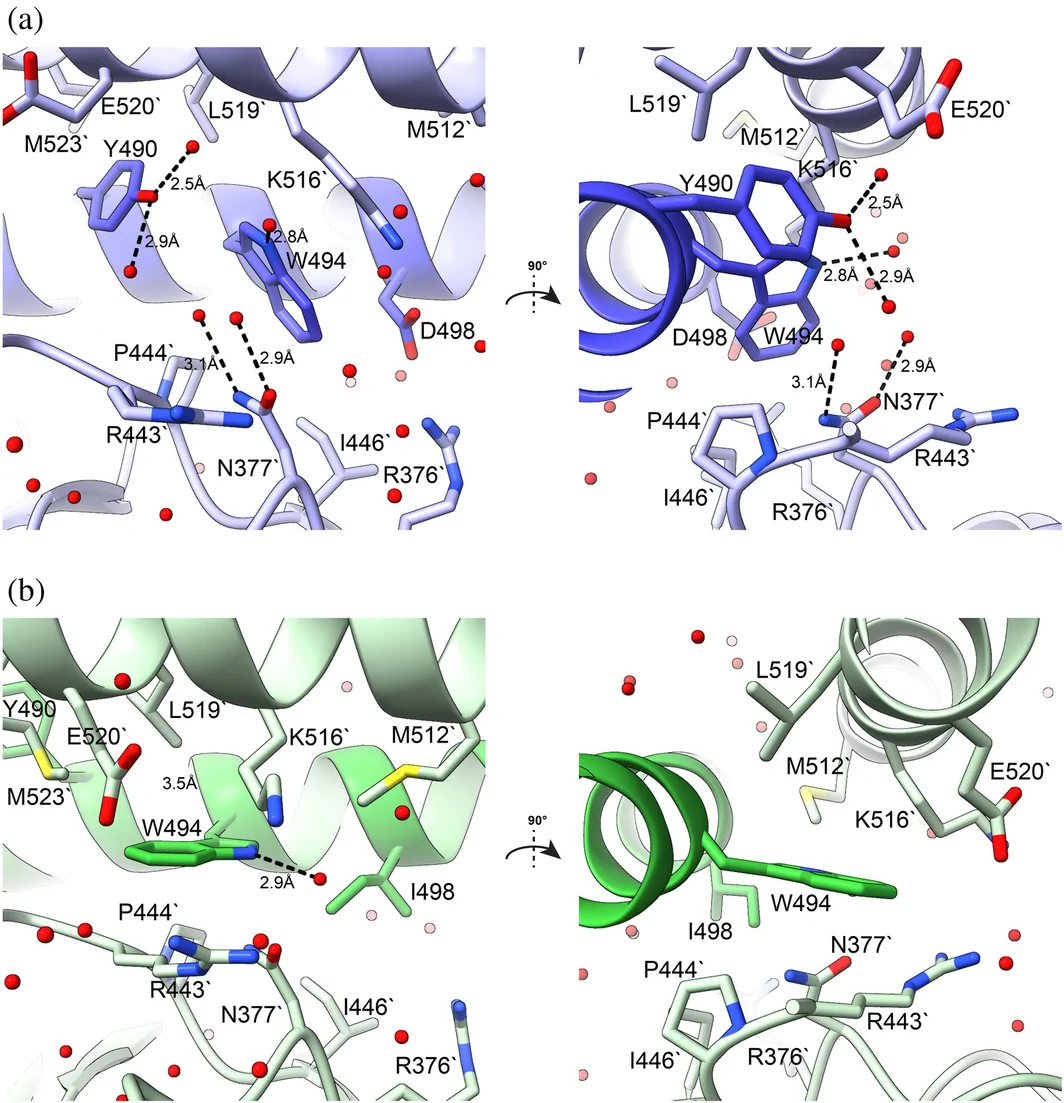
In the SFPQ homodimer, ordered water molecules are present in the pocket at the dimer interface which is occupied by Trp494 in SNS (shown by orthogonal views). (a) Water molecules bound in a pocket present in the SFPQ homodimer (7SP0) between the RRM2′ domain (Arg443′) and α6′ helix (Leu519′) are indicated by hydrogen bonds (black dashes) to key residues. (b) In the SNS homodimer, downward movement of α6′ closes this pocket, which is completely occupied by Trp494.

The greater α6–RRM2 separation in SFPQ appears to be associated with the presence of a greater number of bound water molecules near the core dimer interface than at the SNS interface. To reduce the chance that differences in observed ordered water molecules are not due to different resolution limits, we compared the SNS structure (1.6 Å resolution) with the structure of the SFPQ homodimer that has the highest reported resolution (1.83 Å; 7SP0). In SFPQ, five water molecules occupy the space between Arg443′ (RRM2′) and the α6′ helix above, and are stabilized via hydrogen bonds to Asn377′, Tyr490 and Trp494 (Figure 9a). In contrast, in SNS, this space is completely occupied by the Typ494 indole and the associated shift downward of the α6′ helix (Figure 9b).

In summary, several important molecular rearrangements are associated with a single amino acid substitution at the SNS dimer interface. Compared to the SFPQ homodimer, the Asp498Ile substitution in SNS provides a favorable hydrophobic interaction partner for Ile446′. This is associated with the solvation of Lys516′, and rotation of Trp494 to facilitate its full insertion into the fold of the dimeric partner protein, resulting in a more closed dimer interface and the exclusion of water molecules. This combination of increased hydrophobic complementarity, and maximized incorporation of a bulky aromatic residue coupled to desolvation at the core of the dimer interface is likely to underlie the preference for SFPQ‐NONO heterodimerisation over homodimerisation.

### 2.5 Insights into other DBHS dimer interfaces

**FIGURE 10:**
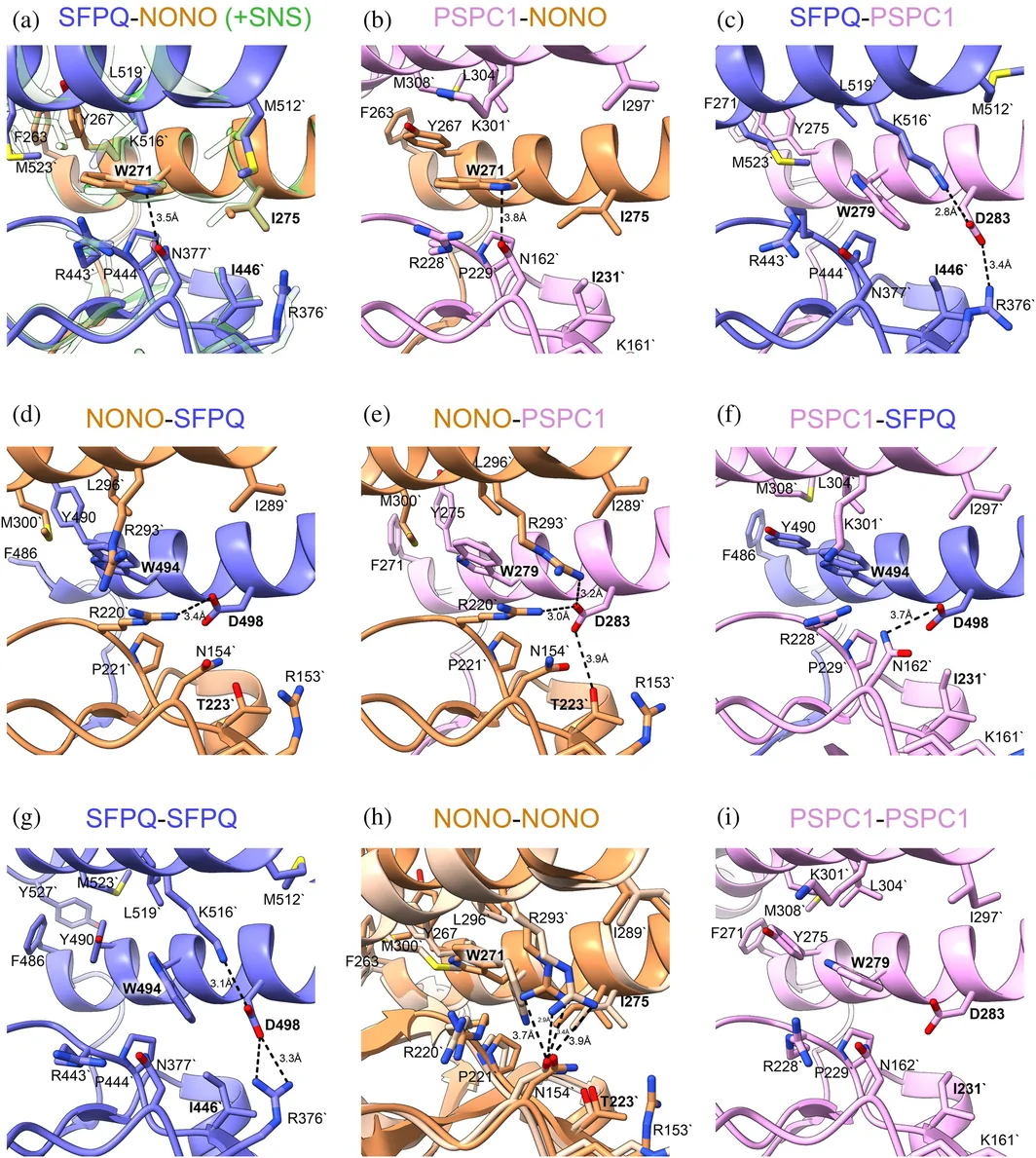
A combination of hydrophobic/polar complementarity and maximized insertion of the core tryptophan residue is likely to contribute to the preference for DBHS proteins to form heterodimers. The core tryptophan and residues of the ‘complementarity site’ (isoleucine, aspartate and/or threonine) are labeled in bold. (a) The dimer interface of SNS (transparent green) replicates the corresponding half of the SFPQ‐NONO heterodimer interface (7LRQ), with all key residues positioned similarly. (b) The key interactions and conformation of the tryptophan (NONO Trp271) are also conserved in the corresponding half of the PSPC1‐NONO heterodimer interface (3SDE). (c–f) All other DBHS heterodimer half‐interfaces, which lack NONO‐Ile275, exhibit a different arrangement of key interface residues, but maintain polar complementarity and/or efficient packing of the core tryptophan indole against the key RRM2 arginine residue (NONO‐Arg220/PSPC1‐Arg228) of the dimeric partner. (g–i) All three DBHS homodimer interfaces lack hydrophobic/polar complementarity, and mostly lack efficient intermolecular packing of the core tryptophan. A partial exception is NONO (h), for which the Trp271 conformation is the same as for the heterodimer interfaces (d–f) in half of the interfaces present in the NONO crystal structure (the asymmetric unit of this crystal structure (5IFM) contains six NONO dimers; in (h), two representative interfaces are shown superposed).

Our engineered DBHS homodimer duplicates one‐half of a heterodimeric interface, resulting in an interface with strict rotational symmetry. Therefore, our study has focussed exclusively on one half of the SFPQ‐NONO dimer interface. Unlike SNS (and native DBHS homodimers) native DBHS heterodimers are asymmetric; that is, they contain two pseudosymmetric ‘half‐interfaces’ (Figure 10). Despite this, insights into DBHS dimerisation preference can be gained by focussing on the corresponding residues in other DBHS interfaces.

Focussing on the Asp498Ile substitution (SFPQ‐Asp498; NONO‐Ile275; PSPC1‐Asp283) highlighted above, NONO is the only DBHS paralogue with an isoleucine at this position; both SFPQ and PSPC1 contain aspartate. Therefore, of the six native heterodimer half‐interfaces, only two contain an isoleucine at this position (i.e. NONO‐Ile275), as for SNS (Figure 10a,b vs. c–f). Comparison of the corresponding region in the SFPQ‐NONO crystal structure shows that all key interface residues are in the same configuration as for SNS (Figure 10a). Specifically, Ile275 of NONO (corresponds to SNS‐Ile498/SFPQ‐Asp498) forms a hydrophobic interaction with Ile446′ of SFPQ, Trp271 (corresponds to Trp494 of SNS/SFPQ) inserts between Arg443′ (RRM2) and Leu519′ (α6), and Lys516′ is solvent‐exposed (in fact, the sidechain is unresolved in this structure). This demonstrates that our construct does indeed reproduce the corresponding half of the SFPQ‐NONO heterodimer interface, as intended. Furthermore, these core interactions are also conserved in the PSPC1‐NONO crystal structure (albeit with a different arrangement of sidechains proceeding towards the distal end of the coiled‐coil) (Figure 10b), consistent with this specific configuration of heterodimeric interface residues being dependent on NONO‐Ile275.

The main residue contributing the complementary hydrophobic interface for NONO‐Ile275 is SFPQ‐Ile446/PSPC1‐Ile231. Importantly, NONO contains a threonine at this position (Thr223), resulting in a loss of hydrophobic complementarity at the NONO homodimer interface (Figure 10h). In the alternate half‐interfaces of SFPQ‐NONO and PSPC1‐NONO, this threonine provides a polar interaction partner for the key aspartate residue in the dimeric partner: SFPQ‐Asp498/PSPC1‐Asp283 (albeit, in the SFPQ‐NONO crystal structure, the donor‐acceptor distance of 4.2 Å is technically too long for a hydrogen bond) (Figure 10d,e). This greater hydrophobic and polar complementarity at this site has been noted previously for the PSPC1‐NONO interface (Knott et al., 2022). Conversely, the fact that the residues at this site are conserved between SFPQ and PSPC1 (i.e. they both contain the residues necessary for complementarity with NONO) precludes polar or hydrophobic complementarity in both half‐interfaces of the SFPQ‐PSPC1 heterodimer (Figure 10c, f).

If this ‘complementarity site’ is the main determinant of DBHS heterodimer preference, it would suggest that NONO‐containing heterodimers are favored over SFPQ‐PSPC1. This is consistent with previous data in Hela cells indicating that PSPC1 predominantly exists as dimers with NONO rather than SFPQ (Fox et al., 2005). However, previous experiments estimating the in vitro binding affinities for SFPQ‐containing dimers found that while both heterodimers exhibited greater affinities than the SFPQ homodimer, SFPQ‐PSPC1 had a six‐fold greater dimerisation affinity than SFPQ‐NONO (Huang et al., 2018). Furthermore, the conservation of non‐complementary residues at the ‘complementarity site’ in SFPQ and PSPC1 means that it cannot serve to explain a preference for heterodimerisation over homodimerisation of these paralogues. Therefore, although chemical complementarity at this site provides an explanation for preferential heterodimerisation in the presence of NONO, the preferential heterodimerisation of SFPQ‐PSPC1 necessitates a different molecular explanation.

Tryptophan and arginine residues are enriched at protein oligomeric interfaces (Bogan & Thorn, 1998; Ofran & Rost, 2003). Indeed, one study identified interacting arginine‐tryptophan pairs as the most favorable amino acid pairing at protein–protein interfaces (Glaser et al., 2001). We note that for the NONO‐containing heterodimers, the indole rings of SFPQ‐Trp494/PSPC1‐Trp279 are rotated ~180° relative to the conformation of NONO‐Trp271 in the alternate half‐interface (Figure 10a, b vs. d, e), maintaining a conformation that inserts between RRM2 (Arg220′) and α6 (Leu296′) of the dimeric partner, maximizing intermolecular interactions in both cases. In addition, SFPQ‐Asp498/PSPC1‐Asp283 provides a complementary electrostatic partner for NONO‐Arg220′; The extended conformation of Arg220′ resulting from this salt‐bridge likely further stabilizes the interface with SFPQ‐Trp494/PSPC1‐Trp279. This heterodimer‐favoring indole conformation is also exhibited by SFPQ‐Trp494 in the SFPQ‐PSPC1 heterodimer (Figure 10f). However, the orientation of the corresponding indole of PSPC1‐Trp279 in the alternate half‐interface (Figure 10c) is the same as the SFPQ homodimer interface (Figure 10g), precluding the intermolecular interaction of this tryptophan with the key arginine in RRM2 (SFPQ‐Arg443′). It may be that the reduced involvement of PSPC1‐Trp279 at the SFPQ‐PSPC1 interface is at least partially compensated for by complimentary electrostatic interactions between PSPC1‐Asp283 and SFPQ‐Arg376′/Lys516′, similar to the SFPQ homodimer.

## 3 DISCUSSION

DBHS proteins are abundant nucleic acid binding proteins that are involved at almost every step of gene regulation and, unsurprisingly, in many pathologies including cancer and neurological diseases (Knott, Bond, & Fox, 2016; Lim et al., 2020; Shadrina et al., 2022; Takeiwa et al., 2024). Previous studies have suggested both paralogue‐specific roles and functional redundancy in different contexts (Li et al., 2014; Major et al., 2019; Mircsof et al., 2015; Naganuma et al., 2012; Sobinoff et al., 2025; Yucel‐Polat et al., 2024), in addition to a dependence on dimeric partner identity for subcellular localisation (Lee et al., 2022), and the possibility of developing therapeutics that target specific paralogues (Florio et al., 2026; Iannuzzi et al., 2025; Kathman et al., 2023; Lindsey et al., 2026). However, interpretation of results from in vivo experiments (especially where the exclusive focus is on a single paralogue) are complicated by the ability of DBHS paralogues to exchange dimeric partners. A molecular understanding of dimer partner preference and the functional consequences of different partner combinations is central to understanding the roles of DBHS proteins in the cell.

DBHS proteins are obligate dimers, existing in solution as homodimers or heterodimers. Therefore, the interface between DBHS dimeric partners must be favorable in both homo‐ and heterodimers. It is therefore unsurprising that a simple description the molecular determinants of heterodimeric preference has remained elusive, with previous structural studies proposing combinations of subtle conformational differences to explain heterodimer preference (Huang et al., 2018; Knott et al., 2022; Schell et al., 2022). Here, we have employed a novel approach to investigate DBHS dimerisation by engineering a chimeric DBHS protein. Our design leverages the two‐fold rotational symmetry of DHBS dimers: by swapping out a 52‐amino acid region responsible for contributing one face of the dimer interface of one DBHS protein (SFPQ) for the corresponding region of another (NONO) we have produced a DBHS homodimer with an interface that duplicates one‐half of the heterodimeric interface. In contrast to native SFPQ and NONO, this engineered chimera shows little detectable exchange with SFPQ under the in vitro conditions tested. Due to the high sequence identity between DBHS proteins in this region, the resulting protein chimera (SNS) differs from native SFPQ at only 14 positions.

Following structure determination, we sought to identify residue‐level determinants of dimer partner preference by comparing the surface area contribution of individual residues to the dimer interface of SNS and SFPQ. Focussing on residues with the greatest difference in contribution to the interface, careful comparison of atomic structures reveals subtle conformational differences that are likely to be involved in determining heterodimeric preference. Strikingly, this analysis suggests that the homodimeric preference of SNS (and by extension the heterodimeric preference of SFPQ‐NONO) may be attributed to a single amino acid substitution. Indeed, of the 14 non‐conserved residues between SFPQ and SNS, the Asp498Ile substitution is the one that is both the least conservative *and* heavily involved at the dimer interface. That is, all other non‐conserved positions are either conservative substitutions, are not at the dimer interface, or are only peripherally involved at the dimer interface (Figures 6 and S10). It is therefore unsurprising that our analysis highlighted residues in spatial proximity to this position. The complexity of the dimer interface urges us to be cautious when attributing large functional effects to single residue substitutions. However, the analysis herein points to the possibility that preferential homodimerisation could be conferred on SFPQ via a single substitution: Asp498Ile. Furthermore, comparison with other DBHS dimer structures (Figure 10) suggests that the core hydrophobic Ile‐Ile interaction introduced by this substitution (native to the SFPQ‐NONO heterodimer) could be recapitulated in both NONO and PSPC1 also via single substitutions: NONO Thr223Ile and PSPC1 Asp283Ile. These are the subject of ongoing experiments.

It is well established that stable protein complexes are characterized by hydrophobic interfaces that exhibit van der Waals and shape complementarity (Janin et al., 2008; Jones & Thornton, 1996). In contrast, buried charged residues tend to destabilize a protein fold, but can be stabilized by the presence of complementary charges (Robinson et al., 2014). A recent large‐scale analysis of human protein complex structures indicated that, while stable, obligate protein–protein interactions are dominated by hydrophobic packing, reversible context‐dependent regulatory interactions rely more heavily on electrostatic complementarity (Grassmann et al., 2023). Our results are consistent with these observations. The charged residue present at the SFPQ homodimer interface (Asp498) engages in strong intermolecular electrostatic interactions (with Lys516′ and Arg376′). The conformational rearrangement required for this electrostatic complementarity comes at the cost of favorable hydrophobic interactions. In SNS (and the corresponding SFPQ‐NONO half‐interface), these electrostatic interactions are exchanged for increased hydrophobic packing, likely resulting in a more stable protein complex. Furthermore, the observed preference for hydrophobic packing at interfaces is inseparable from the fact that interface desolvation is a major contributor to binding energetics, so much so that it can be used as a predictor of the stability of a protein complex (Bogan & Thorn, 1998; Maleki et al., 2013). An in‐depth thermodynamic analysis is outside the scope of this study. Indeed, the dimer exchange experiment (Figures 3 and S3) does not necessarily report on the thermodynamic stability of the SNS homodimer relative to other dimer combinations, as very slow kinetics (kinetic trapping) cannot be ruled out. However, the increased hydrophobic packing, lower water content we observe at the interface, and more favorable calculated complex solvation free energy of SNS compared to the SFPQ homodimer (Table S3) is consistent with the SNS interface being more energetically favorable. In addition, molecular dynamics simulations showed that the SFPQ‐NONO heterodimer overall interaction energy is more favorable than either SFPQ or NONO homodimers (Laurenzi et al., 2022), consistent with the rapid exchange of SFPQ and NONO homodimers to form heterodimers observed herein, and previously (Lee et al., 2022). Given that the SNS homodimer interface recapitulates the SFPQ‐NONO interface, it would be unsurprising if the SNS interface is also more thermodynamically stable than the homodimer interfaces of SFPQ and NONO.

Our focus on a single interface is an obvious limitation of our study. It is almost certain that our analysis has overlooked other subtle contributors to dimer preference. For example, it is possible that the non‐conserved residues in SFPQ and NONO we have excluded from our analysis due to their lack of interface involvement (Figure S10) are contributors to dimer preference in the context of PSPC1. However, our structural comparisons have highlighted the conformation of the core tryptophan residue as a differentiator between DBHS interfaces. With the exception of one half of the SFPQ‐PSPC1 interface, all heterodimer interfaces involve intermolecular packing of this tryptophan with an arginine residue, consistent with the recognized enrichment of tryptophan‐arginine pairs at protein–protein interfaces (Glaser et al., 2001) and the versatility of arginine to form hydrophobic, π‐π and cation‐π interactions (Crowley & Golovin, 2005; Vedad et al., 2024; Vernon et al., 2018). The variable conformation of this tryptophan has been highlighted previously (Passon et al., 2012). Interestingly, a correlation between the sidechain conformation of NONO‐Trp271 and the conformation of the distal coiled‐coil has been observed in an in‐depth crystallographic study on the NONO homodimer (Knott et al., 2022), where two distinct Trp271 conformations were observed in the same crystal (Figure 10h). Although this correlation is likely to be complicated in context of different dimer combinations, this region of the coiled‐coil contains residues that make large contributions to the interface (e.g., SFPQ‐Phe486, Tyr490, Met512, Leu519, M523, Tyr527—see Figure 6) and therefore warrants further investigation. Future studies should also be cautious to consider any possible crystal lattice effects on the conformation of the α6 helix, as highlighted herein.

The feasibility of engineering stable homodimeric DBHS proteins may provide novel avenues for dissecting the non‐redundant roles of all six different DBHS dimeric combinations inside cells. We have shown that, unlike its native counterparts, SNS remains a homodimer in the presence of SFPQ. This result is thus far limited to truncated proteins in vitro; Further experiments are required to investigate whether it is reproducible in cells with full‐length DBHS proteins. In addition, experiments are ongoing to determine whether the SNS homodimer is also stable in the presence of NONO and PSPC1, and to determine the structural integrity of other chimeric permutations resulting from fusing the corresponding regions of all three paralogues. Following from our structural analysis discussed above, we speculate that it may be possible to engineer stable DBHS homodimers via a small number of, or even single, amino acid substitutions. The ability to confer homodimer preference via point mutations may provide a useful tool for deconvoluting the functions of heterodimers and homodimers. Inside cells, the DBHS pool consists of heterodimers and homodimers—the proportions of which depend on relative expression levels and co‐localisation of the different paralogues. Therefore, ascribing the in vivo effects of genetic perturbation of a single DBHS paralogue to loss of homodimer or heterodimer (or both) is a real challenge. For example, NONO loss of function mutations cause neurodevelopmental disease in boys, given the NONO gene resides on the X chromosome (Mircsof et al., 2015). Whether this phenotype is a result of the loss of critical NONO homo‐ or heterodimer function is not known. However, clearly wildtype SFPQ and PSPC1 cannot compensate for this loss of NONO. Individually, SFPQ, NONO and PSPC1 are important for the formation of various nuclear biomolecular condensates, with their flanking IDRs as key contributors to condensation behavior (Hosokawa et al., 2026; Marshall et al., 2023; Shao et al., 2022; Zhang et al., 2022). However, the role of dimeric identity remains unclear in this context also, and has been highlighted previously as a significant confounder to biological interpretation (Marshall et al., 2023). Hence, future experiments in cells where specific heterodimer species are depleted via stabilizing a specific homodimer would allow for the identification of heterodimer‐specific functions. Further, the ability to engineer partner preference also elevates the potential of DBHS proteins as versatile modules for synthetic biology. That is, the native proteins provide modules for driving robust hetero‐interactions; our results demonstrate that these can also be engineered to drive robust homo‐interactions. For example, engineered DBHS proteins, free from cross‐reactivity with native hetero‐preferring domains, could serve as defined modules in split‐reporter reconstitution assays or synthetic transcriptional control circuits, or, given the native role of DBHS proteins as RNA‐binding scaffolds, as building blocks for engineering synthetic RNA‐protein condensates with defined stoichiometry.

Engineered DBHS homodimers may also be useful tools for improving the tractability of future structural studies in vitro. The ready exchange of DBHS dimer partners in solution in the absence of molecular chaperones—observed herein and previously (Koning et al., 2025; Lee et al., 2022)—is remarkable considering the intimate structural arrangement of the DBHS dimer core. It is likely that the emergence of this combinatorial system necessitated the evolution of significant plasticity in the folded dimer core, as suggested by previous crystallographic and solution studies (Hewage et al., 2019; Knott et al., 2022; Lee et al., 2022). It is also likely that this inherent plasticity, combined with the pseudosymmetry of DBHS heterodimers, contributes to solubility and crystallization issues that have made previous structural studies on DBHS proteins challenging (Knott, Panjikar, et al., 2016; Lee et al., 2011; Passon et al., 2011). Indeed, despite the fact that nucleic acid binding is central to their myriad roles in the cell, atomic‐resolution structures of DBHS–nucleic acid complexes remain scarce. Even after extensive optimisation of crystallization conditions, the only reported co‐crystal structure with any nucleic acid to date is of modest resolution (Wang et al., 2022), leaving the molecular basis of nucleic acid recognition by DBHS dimers incompletely understood. Further biophysical characterization is necessary to determine the stability of SNS relative to other DBHS dimer combinations. However, the ease of purification and production of crystals that diffract to atomic resolution, combined with the strict two‐fold symmetry of SNS, presents engineered DBHS proteins as a potentially more tractable approach for future in vitro structural studies aimed at uncovering the molecular details underlying the interaction of DBHS proteins with their myriad nucleic acid targets.

## 4 MATERIALS AND METHODS

### 4.1 Plasmids for heterologous protein expression

A plasmid for the IPTG‐inducible bacterial expression of SFPQ(276–452) + NONO(230–281) + SFPQ(505–535) with an tobacco‐etch virus (TEV) protease N‐terminal hexahistidine tag (His‐tag) was constructed using standard molecular biology techniques (see Table S1). pCDF‐11_SFPQ(276–535) (Lee et al., 2015) was modified by replacement of the DNA sequence encoding residues 453–504 of SFPQ (NCBI accession X70944) with the DNA sequence encoding residues 230–281 of NONO (NCBI accession CCDS14410.1) using Gibson isothermal assembly. The sequence of the expression cassette in the resulting plasmid pCDF‐11_SNS was confirmed by Sanger sequencing. The plasmids used for bacterial expression of His‐tagged SFPQ(276–535), NONO(53–312) and mEGFP‐SFPQ(276–598) have been described previously (pCDF‐11_SFPQ(276–535) (Lee et al., 2015), pET‐Duet‐1_NONO(53–312) (Knott, Panjikar, et al., 2016) and pET28a(+)_mEGFP‐SFPQ(276–598) (Marshall et al., 2023), respectively).

### 4.2 Protein expression and purification

All proteins were expressed in *Escherichia coli* Rosetta 2 (DE3) cells (Novagen) and purified essentially as described previously for DBHS proteins (Knott, Panjikar, et al., 2016; Lee et al., 2015; Marshall et al., 2023).

Briefly, to purify untagged DBHS truncations—SNS, NONO(53–312) and SFPQ(276–535)—His‐tagged protein was isolated from clarified bacterial lysate by Ni^2+^ immobilized metal affinity chromatography (IMAC; 5 mL HisTrap, Cytiva) in a buffer containing 1 M NaCl, 10% glycerol, 50 mM Tris–HCl pH 7.5 and a 25–500 mM imidazole gradient. Peak fractions were pooled, diluted 3‐fold in gel‐filtration buffer (250 mM NaCl, 50 mM L‐proline, 0.5 mM EDTA, 20 mM Tris–HCl, pH 7.5) and subjected to TEV protease digestion overnight at room temperature. The sample was then subjected to Ni^2+^ IMAC again to remove the cleaved His‐tag; the flow‐through, containing untagged protein was collected. For SFPQ(276–535), protein was flash frozen immediately after this step and stored at −80°C until required for partner exchange assays. For SNS and NONO(53–312), the flow‐through from the second Ni^2+^ IMAC was subsequently passed through a HiLoad 16/600 Superdex 200 pg. column (Cytiva) equilibrated in gel‐filtration buffer. Peak fractions were concentrated using a centrifugal device (Amicon) to 4 mg/mL (NONO(53–312)) or 16 mg/mL (SNS). Aliquots were then flash frozen in liquid nitrogen and stored at −80°C.

His‐tagged mEGFP‐SFPQ(276–598) was purified via Ni^2+^ IMAC followed by gel‐filtration chromatography, as described by (Marshall et al., 2023). His‐tagged protein was isolated by passing the clarified bacterial lysate through a 5 mL HisTrap column in binding buffer (1 M KCl, 5% glycerol, 50 mM Tris–HCl, 250 mM L‐arginine, 1 mM PMSF, 10 mM imidazole, pH 7.4), washing the column with ten column volumes of binding buffer followed by ten column volumes wash buffer (same as binding buffer except containing 58 mM imidazole), and eluting bound protein in approximately two column volumes of elution buffer (binding buffer containing 250 mM imidazole). Eluate was subsequently passed through a HiLoad 16/600 Superdex 200 pg. (Cytiva) equilibrated in 0.5 M KCl, 5% glycerol, 1 mM dithiothreitol, 20 mM HEPES pH 7.4. Peak fractions were concentrated to 46 mg/mL, flash frozen and stored at −80°C. All final protein samples exhibited >95% purity, as assessed by SDS‐PAGE (Figure S1).

SDS‐PAGE was performed using 4–20% Mini‐PROTEAN TGX polyacrylamide gels (Bio‐Rad) run at 150–200 V in Tris‐Glycine‐SDS buffer (Astral Scientific). Results were visualized via staining with Coomassie Brilliant Blue.

The concentration of each protein stock was determined via UV absorbance (280 nm) using theoretical extinction coefficients calculated from amino acid composition using ProtParam (Gasteiger et al., 2005), as follows: 11460 M^−1^ for NONO(53–312), 15,930 M^−1^ for SFPQ(276–535), 14,440 M^−1^ for SNS, and 40,800 M^−1^ for mEGFP‐SFPQ(276–598).

### 4.3 Partner exchange affinity chromatography assays

Untagged NONO(53–312), SFPQ(276–535) or SNS were mixed with increasing concentrations of His‐tagged mEGFP‐SFPQ(276–598) and diluted in gel‐filtration buffer (250 mM NaCl, 50 mM L‐proline, 0.5 mM EDTA, 20 mM Tris–HCl pH 7.5). The minimum dilution for any individual protein stock was three‐fold. The final concentration of untagged protein in the mixture was kept constant at 6 μM (DBHS monomer concentration); the concentration of tagged SFPQ was varied to give molar ratios of zero, 0.25, 1 and 4 (i.e. zero, 1.5, 6 and 24 μM). Mixtures were incubated at 37°C for 20 min or 5 h before 400 μL was applied to a 600 μL His SpinTrap column (Cytiva) containing 100 μL of Ni Sepharose resin pre‐equilibrated in binding buffer (1 M KCl, 5% glycerol, 50 mM Tris–HCl, 250 mM L‐arginine, 10 mM imidazole, pH 7.4). The column was inverted to resuspend the resin and centrifuged at 86 × g for 30 s to collect the flow‐through (‘unbound’). The resin was then washed in the same way with 600 μL of binding buffer and bound protein was eluted with 200 μL of elution buffer (binding buffer except containing 250 mM imidazole). ‘Unbound’ and ‘bound’ fractions were analyzed by SDS‐PAGE, performed as described above.

### 4.4 Microscale thermophoresis

The 2′‐α‐fluoro phosphorothioate‐modifed 20‐mer oligonucleotide—IONIS742093—was synthesized with a 5′FAM label by Integrated DNA Technologies (USA), and has been described previously (Knott et al., 2022). Sixteen 1 in 2 serial dilutions of SNS were prepared in MST buffer (250 mM KCl, 50 mM L‐proline, 0.5 mM EDTA, 20 mM Tris–HCl, pH 7.5). An equal volume of 200 nM 5′FAM‐labeled IONIS742093 in MST buffer supplemented with 0.05% polysorbate 20 (Tween® 20) was added to each sample to give final samples with decreasing SNS concentrations 40 μM through 1.22 nM and constant 100 nM IONIS742093, 0.025% polysorbate 20. After mixing, samples were incubated at room temperature for ~10 min before being loaded into standard Monolith capillaries and measuring MST using a Monolith NT.115 (NanoTemper). For all measurements, blue light excitation was used at 40% power; the MST power was set to ‘low’. Experiments were performed in triplicate. Model‐fitting was performed using MO. Affinity Analysis Software.

### 4.5 Crystallization

Protein crystallization was performed by vapor diffusion at 20°C (see Table S2). Initially, protein was subjected to crystallization trials in the presence or absence of polyuracil RNA (U5) (Integrated DNA Technologies) in an attempt to solve structures of the SNS homodimer only and in complex with RNA. Even though no ordered RNA was found to be present in the final crystal structure, the best quality crystals were obtained when grown in the presence of RNA. 12 mg/mL SNS was mixed with 10 mM polyuracil (U5) in a ratio (*v/v*) of 4:1 (final protein concentration of ~10 mg/mL and U5 concentration of 2 mM) immediately prior to setting crystallization trials. Crystals grew as small plates in 15%(*v/v*) Jeffamine ED‐2003, 10% ethanol (MIDASplus condition C1; Molecular Dimensions) in less than one week and were flash frozen in liquid nitrogen using Paratone® N (Hampton Research) as the cryoprotectant.

### 4.6 Diffraction data collection, processing and refinement

X‐ray diffraction data were collected on the MX2 beamline at the Australian Synchrotron (McPhillips et al., 2002) (see Table 1 for details of data collection), processed using *XDS* (Kabsch, 2010) and scaled and merged using *AIMLESS* (Evans & Murshudov, 2013). Structure solution was performed by molecular replacement using *phaser‐MR* (McCoy et al., 2007). The MR search model consisted of chain A of 4wii (SFPQ homodimer) split into three segments: residues 292–367 (RRM1), 368–453 (RRM2), and 454–528 (NOPS‐CC). Refinement of atomic coordinates was done by iterative cycles of automated refinement in *phenix.refine* (Afonine et al., 2012) and manual model building in *Coot* (Emsley et al., 2010) (see Table 2 for refinement statistics). Anisotropic B‐factor refinement was restricted to three automatically defined torsion‐libration‐screws groups, which approximately corresponded to the RRM1, RRM2 and NOPS‐CC domain boundaries, respectively. Final refined atomic coordinates and structure factors can be accessed from the Protein Data Bank (PDB ID 7LRU).

### 4.7 Structural analyses/bioinformatics

All DBHS protein X‐ray crystal structures were extracted from the Protein Data Bank using the advanced search filters: (Lineage Name = “NOPS” AND Annotation Type = “InterPro”) AND Experimental Method = “X‐RAY DIFFRACTION”. Protein–protein interface analyses were performed using the ‘Protein interfaces, surfaces and assemblies’ service *PISA* at the European Bioinformatics Institute (http://www.ebi.ac.uk/pdbe/prot_int/pistart.html) (Krissinel & Henrick,  2007). Buried surface area values for each residue were extracted from *PISA* XML outputs and compared using custom python code written with assistance from Google's Gemini (2.5 Flash) large language model. Protein sequence alignments were performed using Clustal Omega via the EMI‐EMBL webserver (Madeira et al., 2024) and annotated using *ALINE* (Bond & Schüttelkopf, 2009). Molecular graphics and analyses were performed with UCSF ChimeraX (Pettersen et al., 2021).

## AUTHOR CONTRIBUTIONS

**Andrew C. Marshall:** Investigation; funding acquisition; writing – original draft; visualization; writing – review and editing; formal analysis; supervision. **Isabelle Mohnen:** Investigation; writing – review and editing; formal analysis. **Alejandro Villa Gomez:** Investigation; writing – review and editing. **Archa H. Fox:** Conceptualization; writing – review and editing; funding acquisition. **Gavin J. Knott:** Investigation; writing – review and editing; conceptualization. **Charles S. Bond:** Writing – review and editing; conceptualization; funding acquisition; supervision.

## Supporting information


**DATA S1.** “Supplementary Figures.pdf” contains Figures S1–S10. “Supplementary Tables.pdf” contains Tables S1–S3. “MST_SNS_IONIS742093_unmergeddata.pdf” contains raw MST traces, unmerged dose–response data and dissociation constant fitting for each replicate.

**FIGURE S1.** SDS‐PAGE analysis of purified recombinant proteins used in this study. Theoretical molecular weights of each protein are as follows: H6‐GFP‐SFPQ = 67,269, NONO = 30,158, SFPQ = 30,049, SNS = 30,040.
**FIGURE S2.** SNS retains nucleic acid binding ability. The redistribution of fluorescently‐labeled oligonucleotide (IONIS742093; constant concentration) in samples containing increasing concentrations of purified SNS was measured upon subjecting samples to microscale thermophoresis (MST). These data were used to calculate a protein‐nucleic acid binding affinity (*K*_D_) of 105 ± 9 nM. The concentration of protein (x‐axis) refers to the molar concentration of SNS *homodimer*. *N* = 3; error bars are standard deviation.
**FIGURE S3.** SNS does not interact with SFPQ, even after incubation for 5 h.
**FIGURE S4.** Electron density map encompassing part of the coiled‐coil of SNS. A *2Fo‐Fc* map is shown, contoured at 1.4σ. Key interface residues discussed in the text are labeled.
**FIGURE S5.** Superposition of all SFPQ‐containing X‐ray crystal structures (excluding 4WIK, which lacks the RRM1 domain) highlights the variable conformation of RRM1. Alpha‐carbons of residues 370–527 (i.e., excluding RRM1) of both chains in each dimer were defined for superposition. (A) Seven structures contain both RRM1 domains in ‘open' conformation: SFPQ homodimers 4WII (wheat), 6NCQ (blue), 6OWJ (pale green), 7SP0 (pink), 7UK1 (orange); and SFPQ‐NONO heterodimers 6WMZ (gray), 7PU5 (pale blue). (B) Three structures contain RRM1 domains in mixed or intermediate conformations: 4WIJ (homodimer, light blue), 5WPA (SFPQ‐PSPC1, purple), and 7LRQ (SFPQ‐NONO, green). (C) RRM1 domains in the SNS crystal structure (7LRU, yellow) approximate the ‘closed’ conformation—a conformation observed previously only for the crystal structure of SFPQ in complex with RNA (7UJ1, white; RNA is shown as gray ribbons).
**FIGURE S6.** The α6 helix of a symmetry‐related molecule in the SNS crystal occupies part of the RNA‐binding pocket. (A) The surface of the SNS dimer is shown, colored by electrostatic charge. The symmetry‐related α6 helix is shown in magenta cartoon representation. The position of the RNA in the SFPQ‐RNA complex (7UJ1) is overlayed and shown as a white ribbon. Comparison of (B) the SFPQ‐RNA complex (7UJ1) with (C) SNS at the RNA‐binding site shows that the symmetry‐related α6 of SNS (magenta) makes contacts with most of the key RNA‐binding residues. Hydrogen bonds are indicated by black dashes; van der Waals contacts are indicated by transparent gray dashes. Key residues are labeled; residues of the symmetry‐related α6 are in italics.
**FIGURE S7.** Three SFPQ homodimers and SNS colored by BSA (see Figure 5 in main text), highlighting residues involved at the dimer interface.
**FIGURE S8.** The Asp406‐Thr368′ interchain contact in SNS (yellow) is absent in the SFPQ homodimer structures 4WII (wheat), 6NCQ (blue), 6OWJ (pale green). This is due to a shift of Thr368′ (red arrow) associated with the open RRM1 conformation observed in these SFPQ crystal structures.
**FIGURE S9.** The C‐terminal end of the α6′ helix (past ~ Met523′) of SFPQ and SNS is variable in conformation (influenced by crystal packing effects) between different crystal structures. However, the conformation of key residues at the core dimer interface (preceding ~ Met523′) is consistent within different SFPQ homodimer structures, and within different SFPQ‐NONO heterodimer (and SNS) structures. (a) Superposition of SFPQ homodimers (blue; PDBs 4WII, 6NCQ, 6OWJ; five distinct SFPQ chains), SFPQ‐NONO heterodimers (beige; 7LRQ and 7PU5; seven distinct SFPQ chains) and SNS (green) focussed on the C‐terminal half of the SFPQ α6 helix (orthogonal views). The C‐terminus and residues Trp494 and Met523′ of SNS are labeled for reference. (b) Superposition of SFPQ homodimers and SNS at the core dimer interface shows that the conformation of the central Trp494 and surrounding residues is consistent across different SFPQ crystal forms, but distinct from SNS. (c) Superposition SFPQ‐NONO heterodimers and SNS shows that the conformation of the central Trp494 and surrounding residues is consistent across different SFPQ‐NONO crystal forms, and similar to SNS. A notable exception is in the conformation of Lys516′, which points towards the underlying RRM2′ domain in the SFPQ‐NONO heterodimers rather than to solvent in SNS. However, Lys516′ is more solvent‐exposed and flexible in both SFPQ‐NONO and SNS structures (in 7LRQ, the Lys516′ sidechain is absent from the electron density, indicating flexibility) than in the SFPQ homodimer structures, where it forms a salt‐bridge with Asp498. Note: residue numbers in (C) correspond to SNS/SFPQ numbering. i.e., SNS residues 486, 490, 494 and 498 correspond to NONO residues 263, 267, 271 and 275, respectively.
**FIGURE S10.** Aside from Asp498Ile, SFPQ to SNS non‐conservative substitutions are, at most, peripherally involved in the dimer interface. The four non‐conserved residues that contribute some area to the dimer interface (see Figure 6) are colored magenta and shown from two different angles (a and b). SNS is colored green and SFPQ (4WII) is blue; dimeric partners are pale green and pale blue, respectively.

**TABLE S1.** Macromolecule production information.
**TABLE S2.** Experimental details for crystallization of SNS protein.
**TABLE S3.** SNS and SFPQ dimer interface parameters calculated using PISA (Krissinel & Henrick, 2007).

## Data Availability

The data that support the findings of this study are openly available in Protein Data Bank at https://www.rcsb.org/, reference number 7LRU.

## References

[pro70799-bib-0001] Afonine PV , Grosse‐Kunstleve RW , Echols N , Headd JJ , Moriarty NW , Mustyakimov M , et al. Towards automated crystallographic structure refinement with phenix.refine. Acta Crystallogr Biol Crystallogr. 2012;68:352–367. 10.1107/S0907444912001308 PMC3322595 22505256

[pro70799-bib-0002] Anderson P , Kedersha N . RNA granules. J Cell Biol. 2006;172:803–808. 10.1083/jcb.200512082 PMC2063724 16520386

[pro70799-bib-0003] Babu MM , Kriwacki RW , Pappu RV . Versatility from protein disorder. Science. 2012;337:1460–1461. 10.1126/science.1228775 22997313

[pro70799-bib-0004] Bladen CL , Udayakumar D , Takeda Y , Dynan WS . Identification of the Polypyrimidine tract binding protein‐associated splicing factor·p54(nrb) complex as a candidate DNA double‐strand break rejoining factor. J Biol Chem. 2005;280:5205–5210. 10.1074/jbc.M412758200 15590677

[pro70799-bib-0005] Bogan AA , Thorn KS . Anatomy of hot spots in protein interfaces. J Mol Biol. 1998;280:1–9. 10.1006/jmbi.1998.1843 9653027

[pro70799-bib-0006] Bond CS , Schüttelkopf AW . ALINE: a WYSIWYG protein‐sequence alignment editor for publication‐quality alignments. Acta Crystallogr D Biol Crystallogr. 2009;65:510–512. 10.1107/S0907444909007835 19390156

[pro70799-bib-0007] Crowley PB , Golovin A . Cation–π interactions in protein–protein interfaces. Proteins. 2005;59:231–239. 10.1002/prot.20417 15726638

[pro70799-bib-0008] Emsley P , Lohkamp B , Scott WG , Cowtan K . Features and development of Coot. Acta Crystallogr Sect D Biol Crystallogr. 2010;66:486–501. 10.1107/S0907444910007493 PMC2852313 20383002

[pro70799-bib-0009] Evans PR , Murshudov GN . How good are my data and what is the resolution? Acta Crystallogr Biol Crystallogr. 2013;69:1204–1214. 10.1107/S0907444913000061 PMC3689523 23793146

[pro70799-bib-0010] Florio AV , Buré C , Fribourg S . Structural basis for NONO‐specific modification by the α‐chloroacetamide compound (R)‐SKBG‐1. Cell Chem Biol. 2026;33:268–275.e3. 10.1016/j.chembiol.2025.12.013 41519128

[pro70799-bib-0011] Fox AH , Bond CS , Lamond AI . P54nrb forms a heterodimer with PSP1 that localizes to Paraspeckles in an RNA‐dependent manner. Mol Biol Cell. 2005;16:5304–5315. 10.1091/mbc.E05-06-0587 PMC1266428 16148043

[pro70799-bib-0012] Fox AH , Nakagawa S , Hirose T , Bond CS . Paraspeckles: where long noncoding RNA meets phase separation. Trends Biochem Sci. 2018;43:124–135. 10.1016/j.tibs.2017.12.001 29289458

[pro70799-bib-0013] Gasteiger E , Hoogland C , Gattiker A , Duvaud S , Wilkins MR , Appel RD , et al. In: Walker JM , editor. The proteomics protocols handbook. Totowa, NJ: Humana Press; 2005. p. 571–607.

[pro70799-bib-0014] Glaser F , Steinberg DM , Vakser IA , Ben‐Tal N . Residue frequencies and pairing preferences at protein‐protein interfaces. Proteins Struct Funct Genet. 2001;43:89–102. 11276079

[pro70799-bib-0015] Grassmann G , Di Rienzo L , Gosti G , Leonetti M , Ruocco G , Miotto M , et al. Electrostatic complementarity at the interface drives transient protein‐protein interactions. Sci Rep. 2023;13:10207. 10.1038/s41598-023-37130-z PMC10290103 37353566

[pro70799-bib-0016] Hewage TW , Caria S , Lee M . A new crystal structure and small‐angle X‐ray scattering analysis of the homodimer of human SFPQ. Acta Crystallogr Sect F. 2019;75:439–449. 10.1107/S2053230X19006599 PMC6572092 31204691

[pro70799-bib-0017] Hosokawa M , Kawakami R , Imami K , Kurosawa R , Yoshizawa T , Ishihama Y , et al. RNA‐dependent SFPQ condensates coordinate multidimensional regulation of extra‐long neuronal genes. Cell Chem Biol. 2026;33:1146–1164. 10.1016/j.chembiol.2026.06.004 42425084

[pro70799-bib-0018] Huang J , Casas Garcia GP , Perugini MA , Fox AH , Bond CS , Lee M . Crystal structure of a SFPQ/PSPC1 heterodimer provides insights into preferential heterodimerization of human DBHS family proteins. J Biol Chem. 2018;293:6593–6602. 10.1074/jbc.RA117.001451 PMC5925804 29530979

[pro70799-bib-0019] Huang J , Ringuet M , Whitten AE , Caria S , Lim YW , Badhan R , et al. Structural basis of the zinc‐induced cytoplasmic aggregation of the RNA‐binding protein SFPQ. Nucleic Acids Res. 2020;48:3356–3365. 10.1093/nar/gkaa076 PMC7102971 32034402

[pro70799-bib-0020] Iannuzzi CA , Forte IM , Tomeo M , Sfera A , Pagano F , Esposito Abate R , et al. NONO protein regulates the immune response in human triple‐negative breast cancer cells. Int J Mol Sci. 2025;26:8542. 10.3390/ijms26178542 PMC12429829 40943463

[pro70799-bib-0021] Ivanova OM , Anufrieva KS , Kazakova AN , Malyants IK , Shnaider PV , Lukina MM , et al. Non‐canonical functions of spliceosome components in cancer progression. Cell Death Dis. 2023;14:77. 10.1038/s41419-022-05470-9 PMC9895063 36732501

[pro70799-bib-0022] Janin J , Bahadur RP , Chakrabarti P . Protein–protein interaction and quaternary structure. Q Rev Biophys. 2008;41:133–180. 10.1017/S0033583508004708 18812015

[pro70799-bib-0023] Jones S , Thornton JM . Principles of protein‐protein interactions. Proc Natl Acad Sci. 1996;93:13–20. 10.1073/pnas.93.1.13 PMC40170 8552589

[pro70799-bib-0024] Kabsch W . XDS. Acta Crystallogr D Biol Crystallogr. 2010;66:125–132. 10.1107/S0907444909047337 PMC2815665 20124692

[pro70799-bib-0025] Kapoor A , Mondal S , Chaudhary A , Sharma S , Mehra P , Prasad A . A topological review on protein–protein interactions: the development and promises in the era of omics. J Proteins Proteomics. 2024;15:523–544.

[pro70799-bib-0026] Karplus PA , Diederichs K . Assessing and maximizing data quality in macromolecular crystallography. Curr Opin Struct Biol. 2015;34:60–68. 10.1016/j.sbi.2015.07.003 PMC4684713 26209821

[pro70799-bib-0027] Kathman SG , Koo SJ , Lindsey GL , Her H‐L , Blue SM , Li H , et al. Remodeling oncogenic transcriptomes by small molecules targeting NONO. Nat Chem Biol. 2023;19:825–836. 10.1038/s41589-023-01270-0 PMC10337234 36864190

[pro70799-bib-0028] Knott GJ , Bond CS , Fox AH . The DBHS proteins SFPQ, NONO and PSPC1: a multipurpose molecular scaffold. Nucleic Acids Res. 2016;44:3989–4004. 10.1093/nar/gkw271 PMC4872119 27084935

[pro70799-bib-0029] Knott GJ , Chong YS , Passon DM , Liang X‐H , Deplazes E , Conte MR , et al. Structural basis of dimerization and nucleic acid binding of human DBHS proteins NONO and PSPC1. Nucleic Acids Res. 2022;50:522–535. 10.1093/nar/gkab1216 PMC8754649 34904671

[pro70799-bib-0030] Knott GJ , Lee M , Passon DM , Fox AH , Bond CS . Caenorhabditis elegans NONO‐1: insights into DBHS protein structure, architecture, and function. Protein Sci. 2015;24:2033–2043. 10.1002/pro.2816 PMC4815227 26435036

[pro70799-bib-0031] Knott GJ , Panjikar S , Thorn A , Fox AH , Conte MR , Lee M , et al. A crystallographic study of human NONO (p54nrb): overcoming pathological problems with purification, data collection and noncrystallographic symmetry. Acta Crystallogr D Sect Struct Biol. 2016;72:761–769. 10.1107/S2059798316005830 PMC5038197 27303796

[pro70799-bib-0032] Koning HJ , Lai JY , Marshall AC , Stroeher E , Monahan G , Pullakhandam A , et al. Structural plasticity of the coiled–coil interactions in human SFPQ. Nucleic Acids Res. 2024;53:gkae1198. 10.1093/nar/gkae1198 PMC11754644 39698821

[pro70799-bib-0033] Koning HJ , Lai V , Sethi A , Chakraborty S , Ang C‐S , Fox AH , et al. Structural dynamics of IDR interactions in human SFPQ and implications for liquid–liquid phase separation. Acta Crystallogr D Sect Struct Biol. 2025;81:357–379. 10.1107/S2059798325005303 PMC12216677 40574713

[pro70799-bib-0034] Krissinel E , Henrick K . Inference of macromolecular assemblies from crystalline state. J Mol Biol. 2007;372:774–797. 10.1016/j.jmb.2007.05.022 17681537

[pro70799-bib-0035] Kuwahara S , Ikei A , Taguchi Y , Tabuchi Y , Fujimoto N , Obinata M , et al. PSPC1, NONO, and SFPQ are expressed in mouse Sertoli cells and may function as Coregulators of androgen receptor‐mediated Transcription1. Biol Reprod. 2006;75:352–359. 10.1095/biolreprod.106.051136 16641145

[pro70799-bib-0036] Lahaye X , Gentili M , Silvin A , Conrad C , Picard L , Jouve M , et al. NONO detects the nuclear HIV capsid to promote cGAS‐mediated innate immune activation. Cell. 2018;175:488–501.e22. 10.1016/j.cell.2018.08.062 30270045

[pro70799-bib-0037] Lang Y‐D , Jou Y‐S . PSPC1 is a new contextual determinant of aberrant subcellular translocation of oncogenes in tumor progression. J Biomed Sci. 2021;28:57. 10.1186/s12929-021-00753-3 PMC8327449 34340703

[pro70799-bib-0038] Laurenzi T , Palazzolo L , Taiana E , Saporiti S , Ben Mariem O , Guerrini U , et al. Molecular modelling of NONO and SFPQ dimerization process and RNA recognition mechanism. Int J Mol Sci. 2022;23:7626. 10.3390/ijms23147626 PMC9324803 35886974

[pro70799-bib-0039] Lee M , Passon DM , Hennig S , Fox AH , Bond CS . Construct optimization for studying protein complexes: obtaining diffraction‐quality crystals of the pseudosymmetric PSPC1‐NONO heterodimer. Acta Crystallogr D Sect Struct Biol. 2011;67:981–987. 10.1107/S0907444911039606 22101825

[pro70799-bib-0040] Lee M , Sadowska A , Bekere I , Ho D , Gully BS , Lu Y , et al. The structure of human SFPQ reveals a coiled‐coil mediated polymer essential for functional aggregation in gene regulation. Nucleic Acids Res. 2015;43:3826–3840. 10.1093/nar/gkv156 PMC4402515 25765647

[pro70799-bib-0041] Lee PW , Marshall AC , Knott GJ , Kobelke S , Martelotto L , Cho E , et al. Paraspeckle subnuclear bodies depend on dynamic heterodimerisation of DBHS RNA‐binding proteins via their structured domains. J Biol Chem. 2022;298:102563. 10.1016/j.jbc.2022.102563 PMC9643411 36209820

[pro70799-bib-0042] Li S , Li Z , Shu F‐J , Xiong H , Phillips AC , Dynan WS . Double‐strand break repair deficiency in NONO knockout murine embryonic fibroblasts and compensation by spontaneous upregulation of the PSPC1 paralog. Nucleic Acids Res. 2014;42:9771–9780. 10.1093/nar/gku650 PMC4150768 25100870

[pro70799-bib-0043] Lim YW , James D , Huang J , Lee M . The emerging role of the RNA‐binding protein SFPQ in neuronal function and neurodegeneration. Int J Mol Sci. 2020;21:7151. 10.3390/ijms21197151 PMC7582472 32998269

[pro70799-bib-0044] Lindsey GL , Hockley TK , Gomez AV , Marshall AC , Brothers WR , Finney CT , et al. Structural and mechanistic analysis of covalent ligands targeting the RNA‐binding protein NONO. Cell Chem Biol. 2026;33:256–267. 10.1016/j.chembiol.2025.12.010 PMC12918760 41534524

[pro70799-bib-0045] Madeira F , Madhusoodanan N , Lee J , Eusebi A , Niewielska A , Tivey ARN , et al. The EMBL‐EBI job dispatcher sequence analysis tools framework in 2024. Nucleic Acids Res. 2024;52:W521–W525. 10.1093/nar/gkae241 PMC11223882 38597606

[pro70799-bib-0046] Major AT , Hogarth CA , Young JC , Kurihara Y , Jans DA , Loveland KL . Dynamic paraspeckle component localisation during spermatogenesis. Reproduction. 2019;158:267–280. 10.1530/REP-19-0139 31299635

[pro70799-bib-0047] Maleki M , Hall M , Rueda L . Using desolvation energies of structural domains to predict stability of protein complexes. Netw Model Anal Health Inform Bioinforma. 2013;2:267–275.

[pro70799-bib-0048] Marshall AC , Cummins J , Kobelke S , Zhu T , Widagdo J , Anggono V , et al. Different low‐complexity regions of SFPQ play distinct roles in the formation of biomolecular condensates. J Mol Biol. 2023;435:168364. 10.1016/j.jmb.2023.168364 37952770

[pro70799-bib-0049] McCluggage F , Fox AH . Paraspeckle nuclear condensates: global sensors of cell stress? Bioessays. 2021;43:2000245. 10.1002/bies.202000245 33748979

[pro70799-bib-0050] McCoy AJ , Grosse‐Kunstleve RW , Adams PD , Winn MD , Storoni LC , Read RJ . Phaser crystallographic software. J Appl Cryst. 2007;40:658–674. 10.1107/S0021889807021206 PMC2483472 19461840

[pro70799-bib-0051] McPhillips TM , McPhillips SE , Chiu HJ , Cohen AE , Deacon AM , Ellis PJ , et al. Blu‐Ice and the Distributed control system: software for data acquisition and instrument control at macromolecular crystallography beamlines. J Synchrotron Radiat. 2002;9:401–406. 10.1107/s0909049502015170 12409628

[pro70799-bib-0052] Mircsof D , Langouët M , Rio M , Moutton S , Siquier‐Pernet K , Bole‐Feysot C , et al. Mutations in NONO lead to syndromic intellectual disability and inhibitory synaptic defects. Nat Neurosci. 2015;18:1731–1736. 10.1038/nn.4169 PMC5392243 26571461

[pro70799-bib-0053] Näär AM , Lemon BD , Tjian R . Transcriptional coactivator complexes. Annu Rev Biochem. 2001;70:475–501. 10.1146/annurev.biochem.70.1.475 11395415

[pro70799-bib-0054] Naganuma T , Nakagawa S , Tanigawa A , Sasaki YF , Goshima N , Hirose T . Alternative 3′‐end processing of long noncoding RNA initiates construction of nuclear paraspeckles. EMBO J. 2012;31:4020–4034. 10.1038/emboj.2012.251 PMC3474925 22960638

[pro70799-bib-0055] Ofran Y , Rost B . Analysing six types of protein–protein interfaces. J Mol Biol. 2003;325:377–387. 10.1016/s0022-2836(02)01223-8 12488102

[pro70799-bib-0056] Palukuri MV , Patil RS , Marcotte EM . Molecular complex detection in protein interaction networks through reinforcement learning. BMC Bioinformatics. 2023;24:306. 10.1186/s12859-023-05425-7 PMC10394916 37532987

[pro70799-bib-0057] Passon DM , Lee M , Fox AH , Bond CS . Crystallization of a paraspeckle protein PSPC1–NONO heterodimer. Acta Crystallogr Sect F Struct Biol Cryst Commun. 2011;67:1231–1234. 10.1107/S1744309111026212 PMC3212370 22102035

[pro70799-bib-0058] Passon DM , Lee M , Rackham O , Stanley WA , Sadowska A , Filipovska A , et al. Structure of the heterodimer of human NONO and paraspeckle protein component 1 and analysis of its role in subnuclear body formation. Proc Natl Acad Sci. 2012;109:4846–4850. 10.1073/pnas.1120792109 PMC3324020 22416126

[pro70799-bib-0059] Pettersen EF , Goddard TD , Huang CC , Meng EC , Couch GS , Croll TI , et al. UCSF ChimeraX: structure visualization for researchers, educators, and developers. Protein Sci. 2021;30:70–82. 10.1002/pro.3943 PMC7737788 32881101

[pro70799-bib-0060] Rasmussen T , Küspert J , Schönemann L , Geiger D , Böttcher B . The gene‐regulating proteins NONO and SFPQ assemble into ordered filaments. Commun Biol. 2025;9:117. 10.1038/s42003-025-09396-8 PMC12848012 41476260

[pro70799-bib-0061] Robinson AC , Castañeda CA , Schlessman JL , García‐Moreno EB . Structural and thermodynamic consequences of burial of an artificial ion pair in the hydrophobic interior of a protein. Proc Natl Acad Sci U S A. 2014;111:11685–11690. 10.1073/pnas.1402900111 PMC4136602 25074910

[pro70799-bib-0062] Schell B , Legrand P , Fribourg S . Crystal structure of SFPQ‐NONO heterodimer. Biochimie. 2022;198:1–7. 10.1016/j.biochi.2022.02.011 35245601

[pro70799-bib-0063] Seoane B , Carbone A . The complexity of protein interactions unravelled from structural disorder. PLoS Comput Biol. 2021;17:e1008546. 10.1371/journal.pcbi.1008546 PMC7846008 33417598

[pro70799-bib-0064] Shadrina OA , Kikhay TF , Agapkina YY , Gottikh MB . SFPQ and NONO proteins and long non‐coding NEAT1 RNA: cellular functions and role in the HIV‐1 life cycle. Mol Biol. 2022;56:196–209. 10.31857/S0026898422020161 35403619

[pro70799-bib-0065] Shao W , Bi X , Pan Y , Gao B , Wu J , Yin Y , et al. Phase separation of RNA‐binding protein promotes polymerase binding and transcription. Nat Chem Biol. 2022;18:70–80. 10.1038/s41589-021-00904-5 34916619

[pro70799-bib-0066] Shen W , Liang X‐H , Sun H , Crooke ST . 2′‐Fluoro‐modified phosphorothioate oligonucleotide can cause rapid degradation of P54nrb and PSF. Nucleic Acids Res. 2015;43:4569–4578. 10.1093/nar/gkv298 PMC4482069 25855809

[pro70799-bib-0067] Sobinoff AP , Wells JK , Chow M , Nelson CB , Wu X , Cohen SB , et al. NONO, SFPQ, and PSPC1 promote telomerase recruitment to the telomere. Nat Commun. 2025;16:5769. 10.1038/s41467-025-60924-w PMC12218506 40593584

[pro70799-bib-0068] Song X , Sun Y , Garen A . Roles of PSF protein and VL30 RNA in reversible gene regulation. Proc Natl Acad Sci U S A. 2005;102:12189–12193. 10.1073/pnas.0505179102 PMC1189330 16079199

[pro70799-bib-0069] Takeiwa T , Ikeda K , Horie K , Inoue S . Role of RNA binding proteins of the Drosophila behavior and human splicing (DBHS) family in health and cancer. RNA Biol. 2024;21:459–475. 10.1080/15476286.2024.2332855 PMC10984136 38551131

[pro70799-bib-0070] Torres M , Becquet D , Blanchard M‐P , Guillen S , Boyer B , Moreno M , et al. Paraspeckles as rhythmic nuclear mRNA anchorages responsible for circadian gene expression. Nucleus. 2017;8:249–254. 10.1080/19491034.2016.1277304 PMC5499921 28060565

[pro70799-bib-0071] Urban RJ , Bodenburg YH , Wood TG . NH2terminus of PTB‐associated splicing factor binds to the porcine P450scc IGF‐I response element. Am J Physiol Endocrinol Metab. 2002;283:E423–E427. 10.1152/ajpendo.00057.2002 12169434

[pro70799-bib-0072] Vedad J , Bilog M , Chamorro A , Profit AA , Desamero RZB . π‐π stacking interactions mediate molecular recognition between arginine and tryptophan containing peptides derived from human islet polypeptide. J Raman Spectrosc. 2024;55:774–786.

[pro70799-bib-0073] Vernon RM , Chong PA , Tsang B , Kim TH , Bah A , Farber P , et al. Pi‐pi contacts are an overlooked protein feature relevant to phase separation. Elife. 2018;7:e31486. 10.7554/eLife.31486 PMC5847340 29424691

[pro70799-bib-0074] Wang J , Sachpatzidis A , Christian TD , Lomakin IB , Garen A , Konigsberg WH . Insight into the tumor suppression mechanism from the structure of human Polypyrimidine splicing factor (PSF/SFPQ) complexed with a 30mer RNA from murine virus‐like 30S Transcript‐1. Biochemistry. 2022;61:1723–1734. 10.1021/acs.biochem.2c00192 35998361

[pro70799-bib-0075] Widagdo J , Udagedara S , Bhembre N , Tan JZA , Neureiter L , Huang J , et al. Familial ALS‐associated SFPQ variants promote the formation of SFPQ cytoplasmic aggregates in primary neurons. Open Biol. 2022;12:220187. 10.1098/rsob.220187 PMC9516340 36168806

[pro70799-bib-0076] Yucel‐Polat A , Campos‐Melo D , Alikhah A , Strong MJ . Dynamic localization of Paraspeckle components under osmotic stress. Non‐Coding RNA. 2024;10:23. 10.3390/ncrna10020023 PMC11053584 38668381

[pro70799-bib-0077] Zhang S , Cooper JAL , Chong YS , Naveed A , Mayoh C , Jayatilleke N , et al. NONO enhances mRNA processing of super‐enhancer‐associated GATA2 and HAND2 genes in neuroblastoma. EMBO Rep. 2022;24:e54977. 10.15252/embr.202254977 PMC9900351 36416237

