# Supplementary material for: Engineering a protein homodimer from a heterodimer: A chimeric DBHS protein
Source: Protein Sci. 2026 Sep 21;35(10):e70799. doi: 10.1002/pro.70799 (PMC13592225; doi:10.1002/pro.70799)
Supplement: Supplementary file 1 [file PRO-35-e70799-s002.pdf]

Supplementary information

Table S1 Macromolecule production information.

pCDF-11-SNS was constructed by Gibson assembly according to standard protocols. Primers for amplification of overlapping vector and insert are listed 5`-3`

Source organism	*Homo sapiens*
DNA source	pCDF-11-SFPQ(276-535) (Lee *et al.*, 2015) and pDuet-1-NONO(53-312) (Knott *et al.*, 2016)
SFPQ vector fwd primer (aa505)	AGG GAA CAA GTT GAA AAA AAC ATG
SFPQ vector rev primer (aa452)	TTG CTC GAG TGG TTC CAC AAT GAC
NONO insert fwd primer (aa230). NONO sequence is bold.	GTG GAA CCA CTC GAG CAA **TTA GAT GAT GAA GAG GGA C**
NONO insert rev primer (aa281). NONO sequence is bold.	TTT TTC AAC TTg TTC CCT **CTg CTg CTT CTC CAT CTC**
Expression vector	pCDF-11 (EMBL)
Expression host	*Escherichia coli* Rosetta 2 (DE3) (Novagen)
Complete amino acid sequence of the construct produced (after removal of 6×His tag). NONO residues 230-281 are marked in bold.	GAMEGFKANLSLLRRPGEKTYTQRCRLFVG<br>NLPADITEDEFKRLFAKYGEPGEVFINKGKG<br>FGFIKLESRALAEIAKAEL<br>DDTPMRGRQLRVRFATHAAALSVRNLSPYV<br>SNELLEEAFSQFGPIERAVVIVDDRGRSTGK<br>GIVEFASKPAARKAFERCS<br>EGVFLLTTTPRPVIVEPLEQ**LDDEEGLPEK**<br>**LVIKNQQFHKEREQPPRFAQPGSFEYEYAMR**<br>**WKALIEMEKQQ**REQVEKNM<br>KDAKDKLESEMEDAYHEHQANLL

Table S2 Experimental details for crystallization of SNS protein.

Method	Vapor diffusion, sitting drop
Plate type	Art Robbins INTELLI-PLATE 96-3 low vol. reservoir 3-well sitting drop
Temperature (K)	293
Protein concentration	10 mg/mL
Buffer composition of protein solution	20 mM Tris-Cl (pH 7.5), 250 mM NaCl, 50 mM L-Proline and 0.5 mM EDTA, 2 mM polyuracil (U5)
Composition of reservoir solution	15%(*v*/*v*) Jeffamine ED-2003, 10% EtOH
Volume and ratio of drop	400 nL at 1:1 ratio of reservoir:protein solution
Volume of reservoir	60 μL

Table S3 SNS and SFPQ dimer interface parameters calculated using *PISA* (Krissinel & Henrick, 2007).

DBHS homodimer	PDB ID	Total surface area ($Å^2$) ^	# of interface residues	Interface area ($Å^2$)	Interface ΔG (kcal/mol) ^^	# of interface hydrogen bonds	# of interface salt bridges
SNS	7LRU	17931/17931	114/114	4521.9	-60.4	76	18
SFPQ	4WII	17488/17981	121/119	4705.4	-46.1	87	19
SFPQ	6NCQ	18419/18419	115/115	4520.8	-51.6	82	22
SFPQ	6WOJ	18311/18350	115/112	4485.7	-52.5	73	13

^ Total solvent-accessible surface area of each isolate monomer. ^^ Solvation free energy gain upon interface formation; calculated as the difference between the solvation energies of isolated monomers and the dimer (this value excludes electrostatic effects, e.g. intermolecular hydrogen bonds and salt bridges).

Knott, G. J., Panjikar, S., Thorn, A., Fox, A. H., Conte, M. R., Lee, M. & Bond, C. S. (2016). *Acta Crystallographica Section D Structural Biology* **72**, 761–769.

Krissinel, E. & Henrick, K. (2007). *Journal of Molecular Biology* **372**, 774–797.

Lee, M., Sadowska, A., Bekere, I., Ho, D., Gully, B. S., Lu, Y., Iyer, K. S., Trewhella, J., Fox, A. H. & Bond, C. S. (2015). *Nucleic Acids Research* **43**, 3826–3840.
