# Supplementary material for: Engineering a protein homodimer from a heterodimer: A chimeric DBHS protein
Source: Protein Sci. 2026 Sep 21;35(10):e70799. doi: 10.1002/pro.70799 (PMC13592225; doi:10.1002/pro.70799)
Supplement: Supplementary file 2 [file PRO-35-e70799-s003.pdf]

Analysis Report

Analysis Name:	Analysis-Set #1
Type of Analysis:	MST
Evaluation strategy:	Manual
Cold Region Start:	-1 s
Cold Region End:	0 s
Hot Region Start:	0.5 s
Hot Region End:	1.5 s
Exported on:	Fri, 10 Dec 2021 10:19:04 GMT
Software Version:	MO.Affinity Analysis v2.3

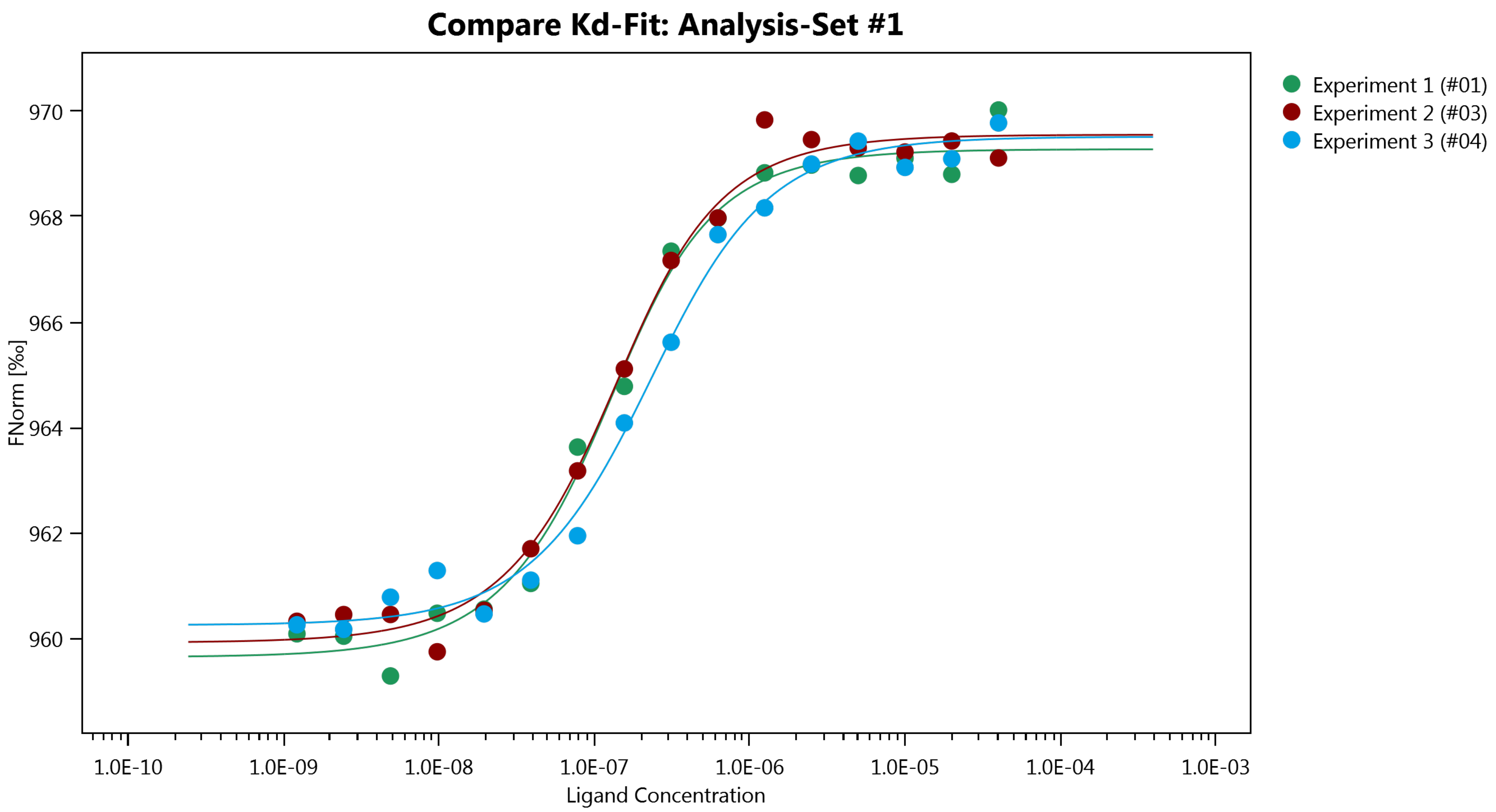

Compare Kd-Fit: Analysis-Set #1
Experiment 1 (#01)
Experiment 2 (#03)
Experiment 3 (#04)
FNorm [‰]
970
968
966
964
962
960
1.0E-10
1.0E-09
1.0E-08
1.0E-07
1.0E-06
1.0E-05
1.0E-04
1.0E-03
Ligand Concentration

Dataset Overview

Name:	Experiment 1 (#01)	Experiment 2 (#03)	Experiment 3 (#04)
Graph Color:	●	●	●
Target Name:	IONIS742093	IONIS742093	IONIS742093
Target Concentration:	100 nM	100 nM	100 nM
Ligand Name:	SNS260	SNS260	SNS260
Ligand Concentration:	40 µM to 0.00122 µM	40 µM to 0.00122 µM	40 µM to 0.00122 µM
n:	1	1	1
Comments:			
Excitation Power:	40%	40%	40%
MST Power:	20%	20%	20%
Temperature:	22.2°C	22.2°C	22.5°C
Kd:	7.426E-08	8.421E-08	1.7886E-07
Kd Confidence:	± 1.3407E-08	± 1.4009E-08	± 2.516E-08
Response Amplitude:	9.6340868	9.6342388	9.2647871
TargetConc:	1E-07[Fixed]	1E-07[Fixed]	1E-07[Fixed]
Unbound:	959.64	959.92	960.25
Bound:	969.28	969.55	969.52
Std. Error of Regression:	0.43721933	0.41361218	0.3782135
Reduced χ^2:			
Signal to Noise:	23.669285	25.020618	26.313122

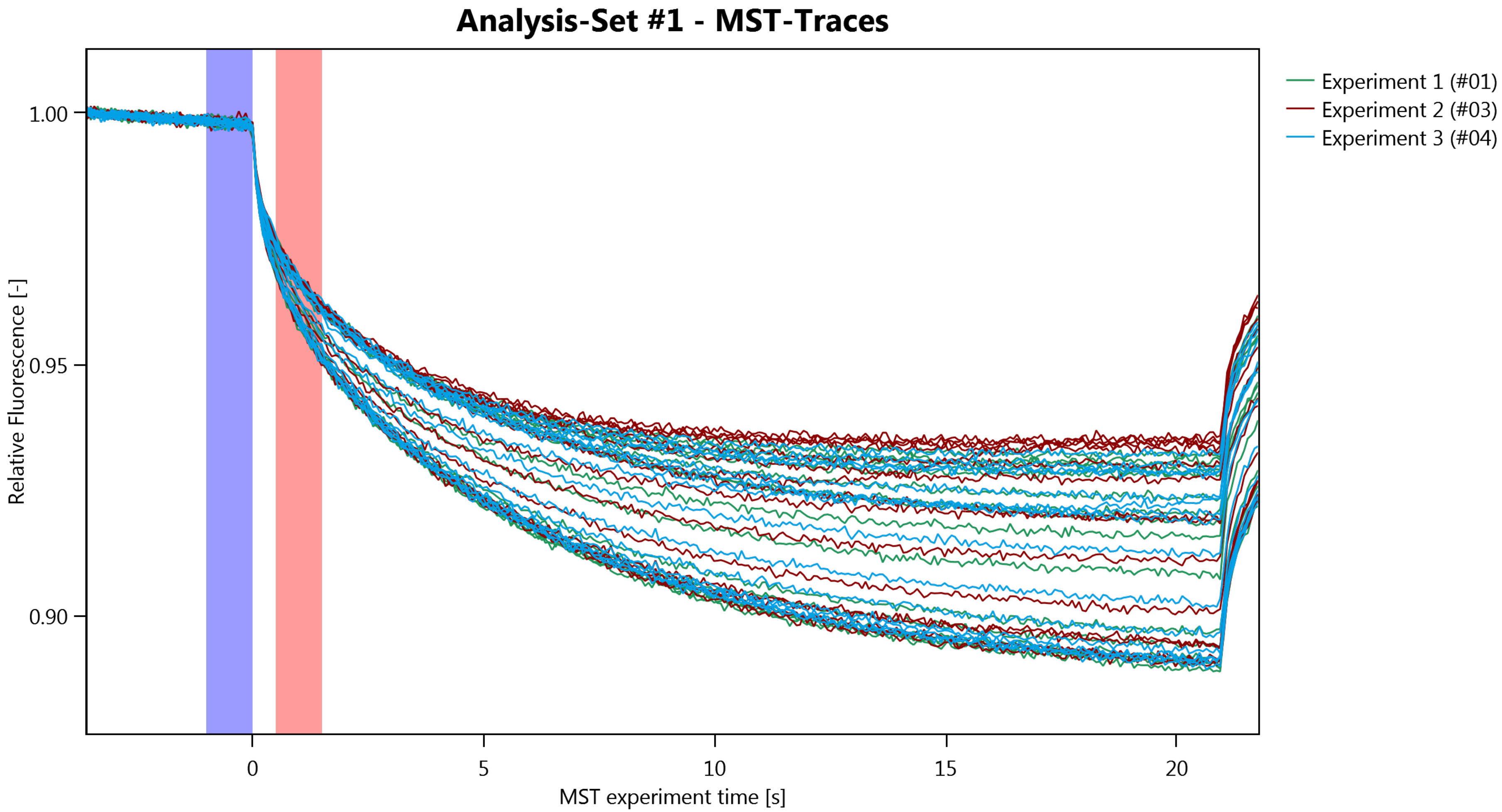
Analysis-Set #1 - MST-Traces
Relative Fluorescence [-]
1.00
0.95
0.90
0
5
10
15
20
MST experiment time [s]
Experiment 1 (#01)
Experiment 2 (#03)
Experiment 3 (#04)

Analysis-Set #1 - Capillary Scan

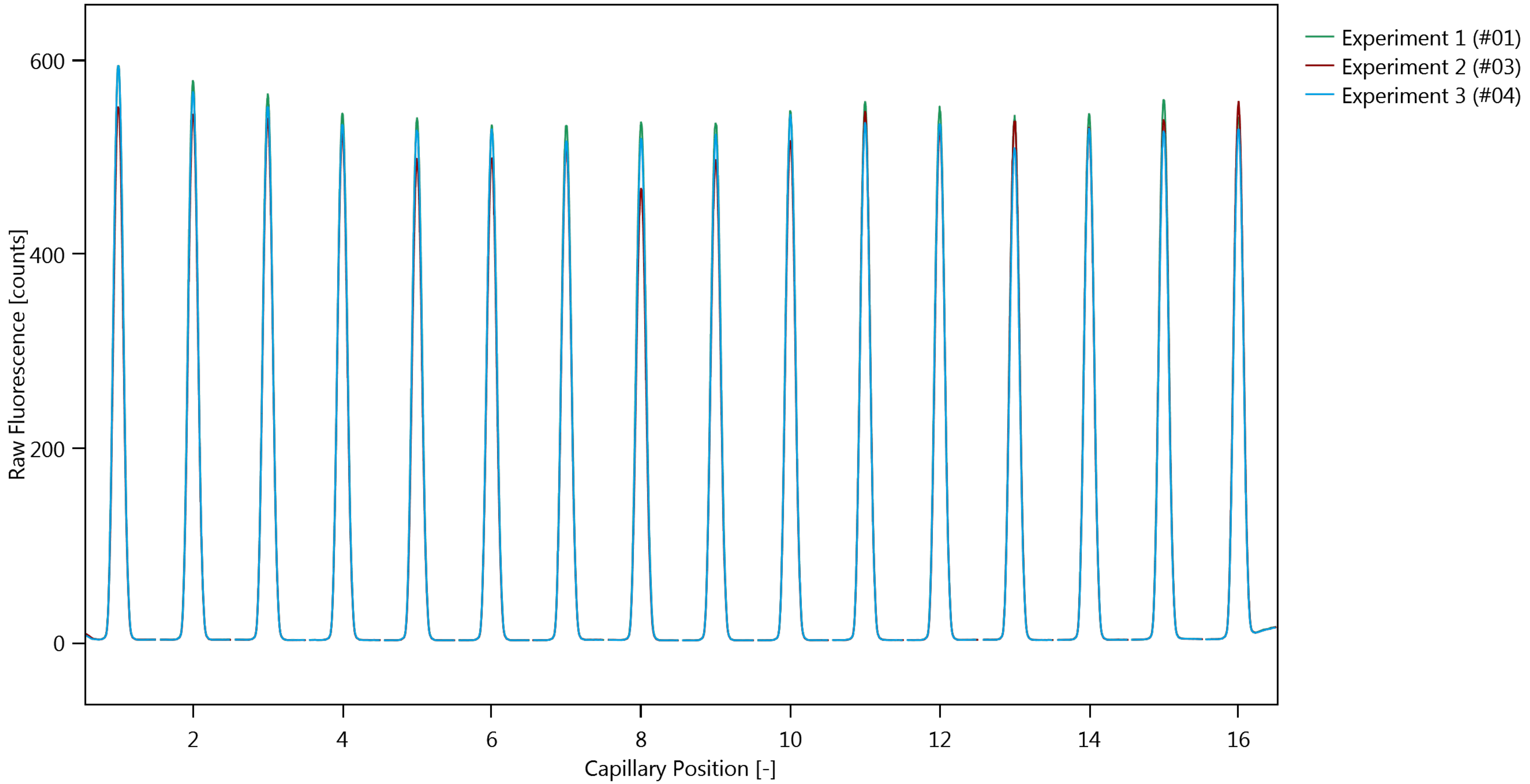

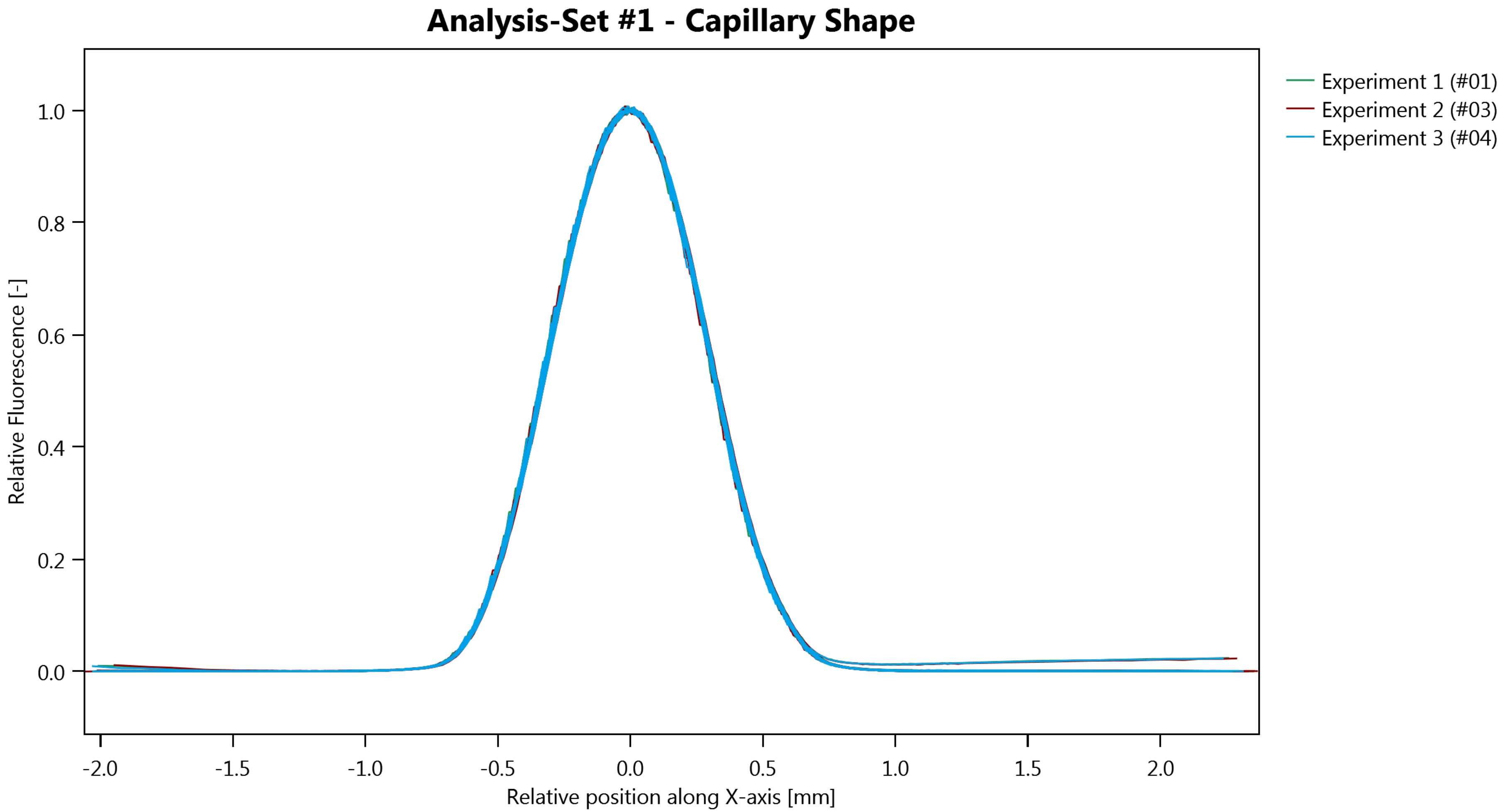
Analysis-Set #1 - Capillary Shape
Experiment 1 (#01)
Experiment 2 (#03)
Experiment 3 (#04)
Relative Fluorescence [-]
1.0
0.8
0.6
0.4
0.2
0.0
-2.0
-1.5
-1.0
-0.5
0.0
0.5
1.0
1.5
2.0
Relative position along X-axis [mm]

Raw data of merged Dose-Response #1

Experiment 1 (#01)

Dose	Response (Average)	Std. Dev.	n
4E-05	970.0222		1
2E-05	968.80202		1
1E-05	969.1073		1
5E-06	968.77987		1
2.5E-06	968.97823		1
1.25E-06	968.83072		1
6.25E-07	967.96919		1
3.125E-07	967.34358		1
1.5625E-07	964.77976		1
7.8125E-08	963.62783		1
3.90625E-08	961.04185		1
1.953125E-08	960.55222		1
9.765625E-09	960.47566		1
4.882812E-09	959.28536		1
2.441406E-09	960.04058		1
1.220703E-09	960.0855		1

Raw data of merged Dose-Response #2

Experiment 2 (#03)

Dose	Response (Average)	Std. Dev.	n
4E-05	969.11142		1
2E-05	969.43418		1
1E-05	969.2257		1
5E-06	969.30473		1
2.5E-06	969.46043		1
1.25E-06	969.83484		1
6.25E-07	967.97385		1
3.125E-07	967.16734		1
1.5625E-07	965.10974		1
7.8125E-08	963.17627		1
3.90625E-08	961.69965		1
1.953125E-08	960.53218		1
9.765625E-09	959.74554		1
4.882812E-09	960.45185		1
2.441406E-09	960.45098		1
1.220703E-09	960.32292		1

Raw data of merged Dose-Response #3

Experiment 3 (#04)

Dose	Response (Average)	Std. Dev.	n
4E-05	969.77718		1
2E-05	969.09651		1
1E-05	968.93348		1
5E-06	969.43205		1
2.5E-06	968.99662		1
1.25E-06	968.16561		1
6.25E-07	967.65859		1
3.125E-07	965.6166		1
1.5625E-07	964.08587		1
7.8125E-08	961.94556		1
3.90625E-08	961.10323		1
1.953125E-08	960.4628		1
9.765625E-09	961.28551		1
4.882812E-09	960.78054		1
2.441406E-09	960.16897		1
1.220703E-09	960.25658		1
