# Supplementary material for: Engineering a protein homodimer from a heterodimer: A chimeric DBHS protein
Source: Protein Sci. 2026 Sep 21;35(10):e70799. doi: 10.1002/pro.70799 (PMC13592225; doi:10.1002/pro.70799)
Supplement: Supplementary file 3 [file PRO-35-e70799-s001.docx]

Supplementary Figures

**Figure S1.** SDS-PAGE analysis of purified recombinant proteins used in this study. Theoretical molecular weights of each protein are as follows: H6-GFP-SFPQ = 67269, NONO = 30158, SFPQ = 30049, SNS = 30040.

**Figure S2**. SNS retains nucleic acid binding ability. The redistribution of fluorescently-labelled oligonucleotide (IONIS742093; constant concentration) in samples containing increasing concentrations of purified SNS was measured upon subjecting samples to microscale thermophoresis (MST). These data were used to calculate a protein-nucleic acid binding affinity (*K*_D_) of 105 ± 9 nM. The concentration of protein (x-axis) refers to the molar concentration of SNS *homodimer*. *N* = 3; error bars are standard deviation.

**Figure S3.** SNS does not interact with SFPQ, even after incubation for 5 hours.

**Figure S4.** Electron density map encompassing part of the coiled-coil of SNS. A *2Fo-Fc* map is shown, contoured at 1.4σ. Key interface residues discussed in the text are labelled.

**Figure S5.** Superposition of all SFPQ-containing X-ray crystal structures (excluding 4WIK, which lacks the RRM1 domain) highlights the variable conformation of RRM1. Alpha-carbons of residues 370-527 (i.e. excluding RRM1) of both chains in each dimer were defined for superposition. (A) Seven structures contain both RRM1 domains in ‘open’ conformation: SFPQ homodimers 4WII (wheat), 6NCQ (blue), 6OWJ (pale green), 7SP0 (pink), 7UK1 (orange); and SFPQ-NONO heterodimers 6WMZ (grey), 7PU5 (pale blue). (B) Three structures contain RRM1 domains in mixed or intermediate conformations: 4WIJ (homodimer, light blue), 5WPA (SFPQ-PSPC1, purple), and 7LRQ (SFPQ-NONO, green). (C) RRM1 domains in the SNS crystal structure (7LRU, yellow) approximate the ‘closed’ conformation – a conformation observed previously only for the crystal structure of SFPQ in complex with RNA (7UJ1, white; RNA is shown as grey ribbons).

**Figure S6.** The α6 helix of a symmetry-related molecule in the SNS crystal occupies part of the RNA-binding pocket. (A) The surface of the SNS dimer is shown, coloured by electrostatic charge. The symmetry-related α6 helix is shown in magenta cartoon representation. The position of the RNA in the SFPQ-RNA complex (7UJ1) is overlayed and shown as a white ribbon. Comparison of (B) the SFPQ-RNA complex (7UJ1) with (C) SNS at the RNA-binding site shows that the symmetry-related α6 of SNS (magenta) makes contacts with most of the key RNA-binding residues. Hydrogen bonds are indicated by black dashes; van der Waals contacts are indicated by transparent grey dashes. Key residues are labelled; residues of the symmetry-related α6 are in italics.

**Figure S7.** Three SFPQ homodimers and SNS coloured by BSA (see Figure 5 in main text), highlighting residues involved at the dimer interface.

**Figure S8.** The Asp406-Thr368` interchain contact in SNS (yellow) is absent in the SFPQ homodimer structures 4WII (wheat), 6NCQ (blue), 6OWJ (pale green). This is due to a shift of Thr368` (red arrow) associated with the open RRM1 conformation observed in these SFPQ crystal structures.

**Figure S9.** The C-terminal end of the α6` helix (past ~ Met523`) of SFPQ and SNS is variable in conformation (influenced by crystal packing effects) between different crystal structures. However, the conformation of key residues at the core dimer interface (preceding ~ Met523`) is consistent within different SFPQ homodimer structures, and within different SFPQ-NONO heterodimer (and SNS) structures. (**A**) Superposition of SFPQ homodimers (blue; PDBs 4WII, 6NCQ, 6OWJ; five distinct SFPQ chains), SFPQ-NONO heterodimers (beige; 7LRQ and 7PU5; seven distinct SFPQ chains) and SNS (green) focussed on the C-terminal half of the SFPQ α6 helix (orthogonal views). The C-terminus and residues Trp494 and Met523` of SNS are labelled for reference. (**B**) Superposition of SFPQ homodimers and SNS at the core dimer interface shows that the conformation of the central Trp494 and surrounding residues is consistent across different SFPQ crystal forms, but distinct from SNS. (**C**) Superposition SFPQ-NONO heterodimers and SNS shows that the conformation of the central Trp494 and surrounding residues is consistent across different SFPQ-NONO crystal forms, and similar to SNS. A notable exception is in the conformation of Lys516`, which points towards the underlying RRM2` domain in the SFPQ-NONO heterodimers rather than to solvent in SNS. However, Lys516` is more solvent-exposed and flexible in both SFPQ-NONO and SNS structures (in 7LRQ, the Lys516` sidechain is absent from the electron density, indicating flexibility) than in the SFPQ homodimer structures, where it forms a salt-bridge with Asp498. Note: residue numbers in (C) correspond to SNS/SFPQ numbering. i.e. SNS residues 486, 490, 494 and 498 correspond to NONO residues 263, 267, 271 and 275, respectively.

**Figure S10.** Aside from Asp498Ile, SFPQ to SNS non-conservative substitutions are, at most, peripherally involved in the dimer interface. The four non-conserved residues that contribute some area to the dimer interface (see Fig. 5) are coloured magenta and shown from two different angles (A and B). SNS is coloured green and SFPQ (4WII) is blue; dimeric partners are pale green and pale blue, respectively.
